# Growth, Antioxidant, and Physiological Responses Associated with Tuber Yield in Cassava Under Irrigation and Late-Season Drought Conditions in a Tropical Savanna Climate

**DOI:** 10.3390/plants15193003

**Published:** 2026-09-30

**Authors:** Supranee Santanoo, Passamon Ittipong, Nimitr Vorasoot, Sanun Jogloy, Kochaphan Vongcharoen, Piyada Theerakulpisut, Poramate Banterng

**Affiliations:** 1 Department of Agronomy, Faculty of Agriculture, Khon Kaen University, Khon Kaen 40002, Thailand; suprsa@kku.ac.th (S.S.); passamon.i@kkumail.com (P.I.); nvorasoot1@gmail.com (N.V.); sjogloy@gmail.com (S.J.); 2 Faculty of Science and Health Technology, Kalasin University, Kalasin 46000, Thailand; kocha_9@hotmail.com; 3 Department of Biology, Faculty of Science, Khon Kaen University, Khon Kaen 40002, Thailand; piythe@kku.ac.th; 4 Plant Breeding Research Center for Sustainable Agriculture, Faculty of Agriculture, Khon Kaen University, Khon Kaen 40002, Thailand

**Keywords:** *Manihot esculenta*, drought stress, carbon partitioning, assimilate remobilization, antioxidant activity, photosynthetic performance, source-sink dynamics, storage root yield

## Abstract

In tropical savanna climates such as the Northeast of Thailand, cassava is cultivated year-round but frequently experiences drought during storage root development, reducing productivity. However, the physiological mechanisms linking drought responses to carbon allocation and yield in Thai cassava cultivars remain poorly understood. In this field study, two cassava genotypes, including RY72 and CMR35–91–63, were planted during the late hot season (May 2022) and grown under either continuous irrigation (control) or drought conditions imposed for 60 days from 6 to 8 months after planting (MAP) during the dry season (November 2022 to January 2023), with no irrigation. Following the drought treatment, the plants were rewatered and allowed to grow until harvest at 12MAP. Moderate drought had a greater impact on carbon allocation and biomass accumulation than on photosynthetic capacity. Both genotypes maintained photosystem II efficiency, and photosynthetic performance under drought while enhancing antioxidant enzymes activity, thereby limiting oxidative damage and enabling rapid recovery after rewatering. During drought, photoassimilates were preferentially translocated to tuber in RY72 but to above-ground organs in CMR35–91–63. Following rewatering, RY72 hadhigher net photosynthesis rate and maintained higher sink activity of the tubers resulting in higher tuber biomass and starch content at final harvest. Therefore, stable photosynthesis performance together with high sink activity of storage roots during drought and after recovery are important characters alleviating yield loss in cassava subjected to late-season drought.

## 1. Introduction

Cassava (*Manihot esculenta* Crantz) is one of the most important tropical root crops, serving as a major source of carbohydrates for human consumption and as a strategic feedstock for starch, animal feed, pharmacological products, bioethanol, and numerous bio-based industries [1,2]. Owing to its high starch productivity and adaptability to low-rainfall areas, cassava has become increasingly important for ensuring food security and supporting the bio-economy under changing climatic conditions [3,4,5]. Thailand is one of the world’s largest cassava producers (28.0 million tons) and the second largest exporter globally, with cassava exports valued at $518 million in 2024 [6,7]. Production is concentrated in the northeastern region, which accounts for 54% of the national harvested area, where cassava cultivation relies almost entirely on rainfed agriculture [8]. Consequently, seasonal drought remains the principal environmental constraint limiting Thai cassava productivity and starch yield [9,10]. Although cassava is widely recognized as a drought-tolerant crop, prolonged water deficit substantially reduces biomass accumulation and storage root development [11,12,13]. In Thailand, drought frequently occurs during the mid-to-late growing season, coinciding with the critical starch accumulation phase (approximately 6–8 months after planting). Cassava has three main growth stages before harvest (10–12 months): (1) leaf and root development (0–3 months), (2) stem and leaf growth (3–6 months), and (3) carbohydrate translocation and storage root bulking (6–10 months) [5]. The third stage is critical for yield and starch accumulation, as water limitation restricts carbon assimilation and alters source-sink relationships, ultimately reducing storage root yield and starch content [14,15]. However, these responses to water limitation vary among cassava genotypes, reflecting differences in their genetic potential for maintaining yield performance under drought conditions [16,17]. Rayong72 (RY72) is specifically recommended for its high drought tolerance, whereas other cultivars (Kasetsart50, Rayong9, Rayong5, Huay Bong80 and Phirun1) and lines (CMR35–91–63 and CMR38–125–77) exhibit strong adaptability, rapid growth, and high yields [18,19]. Ultimately, the selection of these cultivars depends on regional soil conditions and rainfall [18]. As climate change is expected to increase the frequency and severity of drought events, understanding the physiological mechanisms, including photosynthesis, carbohydrate partitioning, and antioxidant activity, that enable cassava to maintain productivity under water-limited conditions is becoming increasingly important for sustaining cassava production in northeastern Thailand.

Photosynthetic performance is one of the primary determinants of cassava productivity under drought. Water deficit induces stomatal closure, reduces leaf CO_2_ assimilation, impairs photosystem II efficiency, and decreases water use efficiency [20,21]. These responses influence carbon fixation and consequently modify carbohydrate partitioning among leaves, stems, and storage roots [15,22,23]. Because starch accumulation in cassava depends largely on efficient transport of sucrose from source leaves to sink organs, drought-induced disruption of carbohydrate allocation can substantially limit storage root development [15,24]. In addition, drought stress promotes excessive production of reactive oxygen species (ROS), leading to oxidative damage to cellular structures [25,26]. Therefore, drought tolerance is governed by the coordinated regulation of photosynthesis, carbon allocation, antioxidant metabolism, and storage root sink activity rather than by any single physiological trait [22,27].

Previous studies have examined drought-induced changes in photosynthesis, water use efficiency, carbohydrate partitioning, and yield formation in cassava [15,28,29]. However, these physiological processes, especially antioxidant activity, have rarely been studied during the late growth stage (storage root starch accumulation), a critical determinant of final yield. Moreover, the physiological mechanisms underlying drought adaptation in Thai tropical cassava cultivars remain poorly understood, particularly the coordination of photosynthetic efficiency, carbohydrate translocation, starch accumulation, antioxidant responses, and yield maintenance during the late growth stage. Elucidating the relationships among these physiological traits and storage root development will provide a mechanistic understanding of drought adaptation in cassava. Such knowledge is essential for identifying physiological traits that contribute to yield stability under water deficit and for improving cultivar selection for rainfed production systems [30]. The findings will also contribute to breeding programs by identifying physiological markers associated with superior drought tolerance and efficient carbon utilization. Ultimately, these advances will support sustainable cassava production, improve starch yield under increasingly variable climatic conditions, and strengthen the resilience of Thailand’s cassava industry.

## 2. Results

### 2.1. Photosynthetic Performance

**Figure 1:**
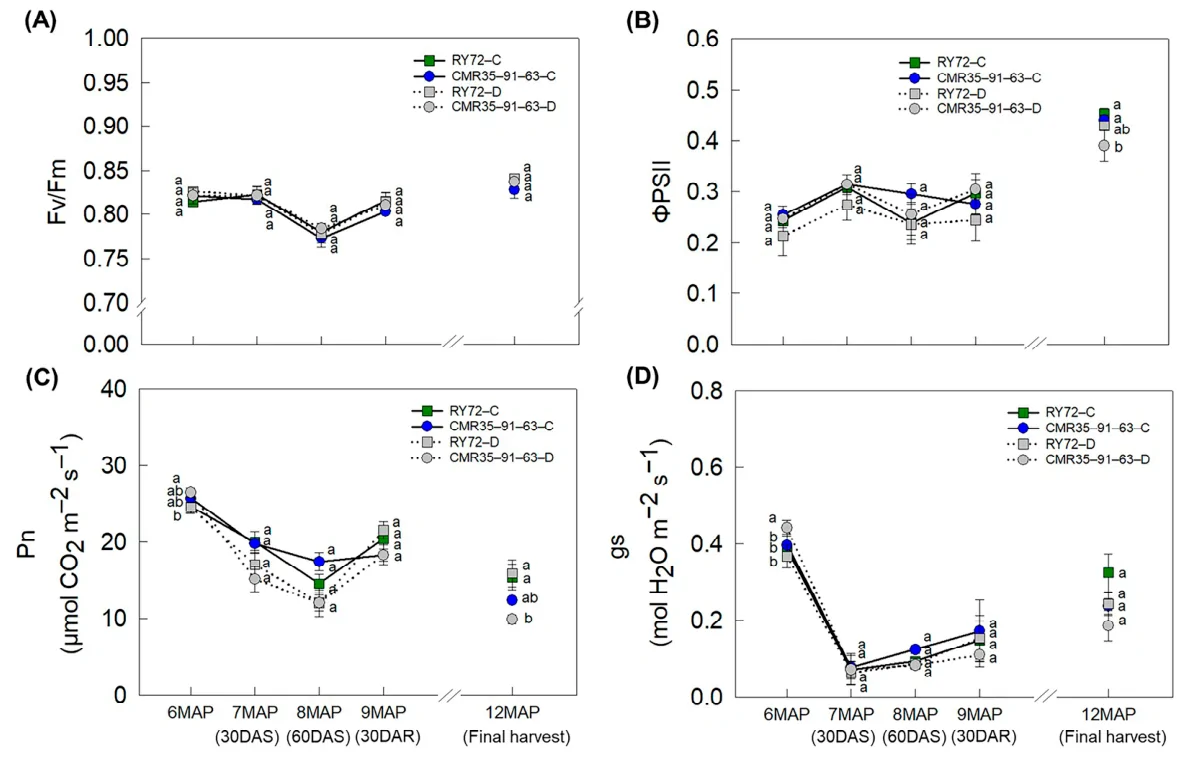
Photosynthetic parameters, including the maximum quantum yield of PSII (Fv/Fm, (**A**)), effective quantum yield of PSII photochemistry (ΦPSII, (**B**)), net photosynthesis (Pn, (**C**)), and stomatal conductance (gs, (**D**)) of RY72 and CMR35–91–63 measured at 6, 7, 8, 9, and 12 months after planting (MAP). Plants were grown under well-watered conditions (continuous irrigation from planting to 12MAP) and drought conditions (irrigation withheld for 60 days during the dry season from 6 to 8MAP, followed by rewatering until 12MAP). Different lowercase letters indicate significant differences (*p* < 0.05 or *p* < 0.01) among water conditions and genotypes within each crop age. Data are presented as mean ± SE (n = 4).

Chlorophyll fluorescence parameters, including maximum quantum yield of PSII (Fv/Fm) and effective quantum yield of PSII (ΦPSII), and leaf gas exchange were evaluated in cassava genotypes RY72 and CMR35–91–63 under different water regimes (Figure 1; Table S1). Overall, drought stress (30 and 60 days after stress, DAS) during the late growth stage at 7 to 8MAP showed no significant effects on Fv/Fm, ΦPSII, Pn, and gs. In contrast, Fv/Fm declined significantly (*p* < 0.05) with crop age in both genotypes (Tables S1 and S2), reaching the lowest values at 8MAP (0.778–0.789 in RY72 and 0.773–0.784 in CMR35–91–63) (Figure 1A). The ΦPSII remained relatively stable under drought, indicating that photosynthetic efficiency was largely maintained (Figure 1B). Both parameters reached their highest values at 12MAP, with ΦPSII ranging from 0.390 to 0.453. At 12MAP, drought significantly (*p* < 0.05) reduced ΦPSII by approximately 11% in CMR35–91–63 (0.390 vs. 0.440 in the control), whereas no significant reduction was observed in RY72 (0.429 vs. 0.453).

Leaf gas exchange parameters, including net photosynthesis rate (Pn) and stomatal conductance (gs), were not significantly affected by drought (30 and 60DAS) during the late growth stage (7–8MAP), consistent with the stable chlorophyll fluorescence parameters (Figure 1C,D; Tables S2 and S8). Across both water regimes, 6MAP plants exhibited the highest Pn and gs, whereas Pn declined with crop age and reached the lowest values at 12MAP. Under the well-watered conditions, Pn was maximum at 6MAP in RY72 (24.56 µmol CO_2_ m^−2^ s^−1^) and CMR35–91–63 (25.60 µmol CO_2_ m^−2^ s^−1^), declining to 15.33 and 12.31 µmol m^−2^ s^−1^ at 12MAP, respectively. Although drought did not significantly reduce Pn, numerical reductions of 15–17% in RY72 and 24–31% in CMR35–91–63 were observed relative to the well-watered plants. After 30 days of rewatering, Pn recovered significantly (*p* < 0.05) to 21.50 and 18.29 µmol m^−2^ s^−1^ in RY72 and CMR35–91–63, respectively. For the drought-treated plants at 12MAP, Pn remained significantly (*p* < 0.05) higher in RY72 (15.38 µmol CO_2_ m^−2^ s^−1^) than in CMR35–91–63 (9.86 µmol CO_2_ m^−2^ s^−1^). The gs showed patterns similar to Pn for both the well-watered and drought conditions, with maximum values at 6MAP, dramatically dropped (*p* < 0.05) at 7MAP, remained low at 8MAP, coinciding with low temperature and limited rainfall (Table 1), and slightly recovered at 9MAP. Under drought, gs decreased by 6–11% in RY72 and 7–33% in CMR35–91–63. At 12MAP, gs increased but remained lower than the well-watered conditions.

### 2.2. Effects of Drought Stress on Carbohydrate Partitioning

#### 2.2.1. Changes in Reducing Sugar (RS), Non-Reducing Sugar (NRS), Total Sugar, and Starch Concentrations in Cassava Leaves

**Figure 2:**
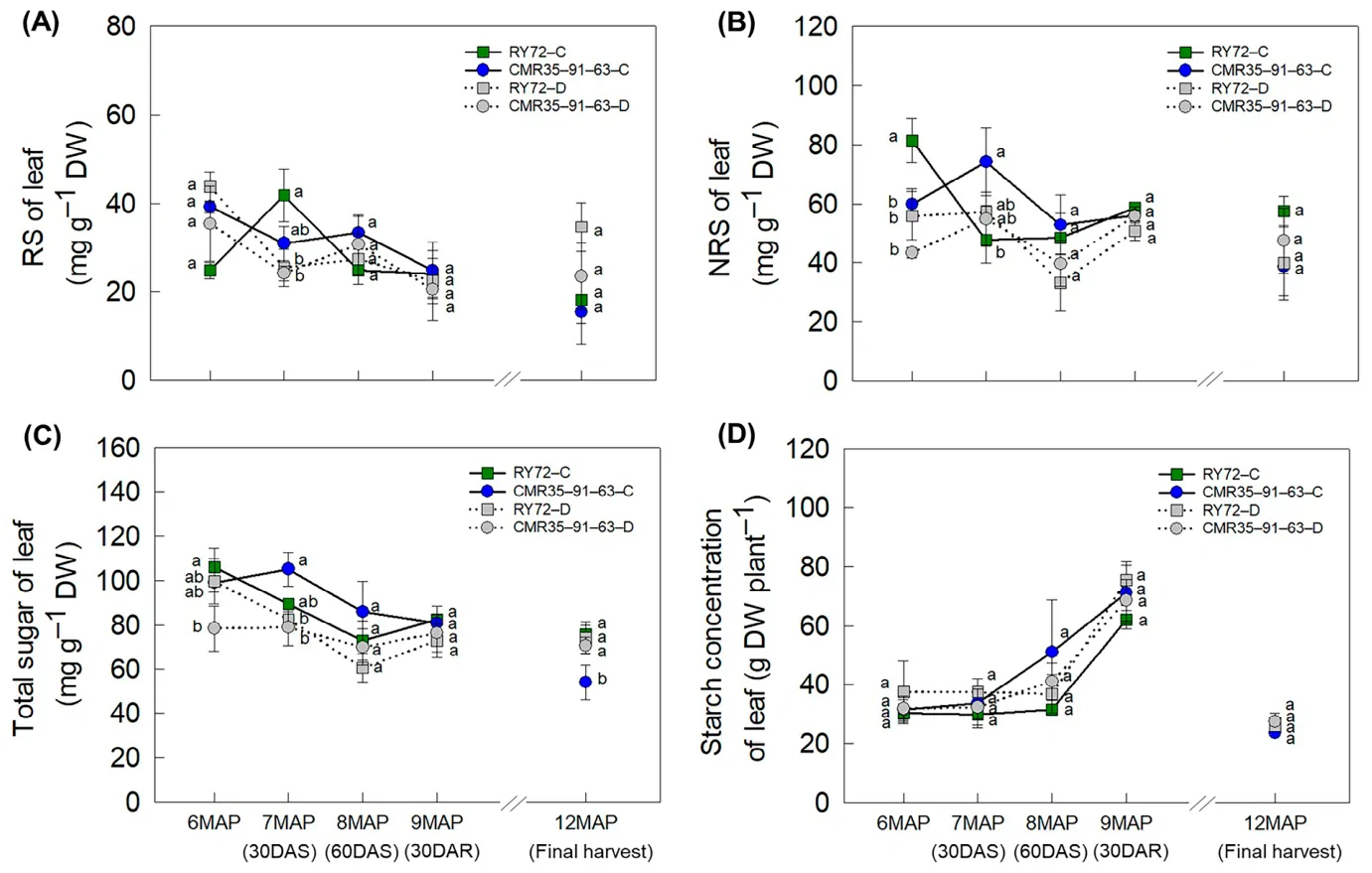
Leaf reducing (**A**), non-reducing (**B**) and total sugar (**C**)) and starch concentrations (**D**) of RY72 and CMR35–91–63 measured at 6, 7, 8, 9, and 12 months after planting (MAP). Plants were grown under well-watered conditions (continuous irrigation from planting to 12MAP) and drought conditions (irrigation withheld for 60 days during the dry season from 6 to 8MAP, followed by rewatering until 12MAP). Different lowercase letters indicate significant differences (*p* < 0.05 or *p* < 0.01) among water conditions and genotypes within each crop age. Data are presented as mean ± SE (n = 4).

Leaf soluble sugars were influenced primarily by crop age rather than drought (Figure 2; Table S3). Under well-watered conditions, reducing sugar (RS) varied significantly with crop age, ranging from 18.14–41.89 mg g^−1^ DW in RY72 and 15.37–39.27 mg g^−1^ DW in CMR35–91–63. The highest RS occurred at 7MAP in RY72 and 6MAP in CMR35–91–63, whereas the lowest values were recorded at 12MAP. Drought significantly (*p* < 0.05) reduced RS only in RY72 at 30DAS (−39%), while CMR35–91–63 showed a non-significant reduction (−22%). No significant differences between water regimes were detected at 60DAS, after rewatering, and at 12MAP, although drought-treated plants tended to have higher RS than the controls at the final harvest.

Non-reducing sugar (NRS) concentrations were generally higher than RS concentrations across all crop ages. Under the well-watered conditions, RY72 showed the highest NRS at 6MAP (81.45 mg g^−1^ DW), whereas CMR35–91–63 showed its highest NRS concentration at 7MAP (74.36 mg g^−1^ DW). Drought caused a 25 to 31% reduction in NRS concentration, except for a 20% increase in RY72 at 7MAP; however, these responses were not statistically significant. After rewatering, NRS in RY72 remained lower than the control (50.68 vs. 58.70 mg g^−1^ DW), whereas CMR35–91–63 recovered to the control level. At 12MAP, NRS were not significantly different between water regimes and were lower than those in the rewatered plants.

Leaf total sugar showed a pattern similar to NRS. Under the well-watered conditions, the highest total sugar was observed at 6MAP in RY72 (106.14 mg g^−1^ DW), while the highest concentration in CMR35–91–63 was recorded at 7MAP (105.20 mg g^−1^ DW). Drought reduced total sugar by 8–25%, with a significant reduction (–25%) in CMR35–91–63 at 30DAS (7MAP). After rewatering, at 9MAP, total sugar in both genotypes slightly increased but remained lower than the respective controls (73.01 vs. 82.63 mg g^−1^ DW in RY72 and 76.35 vs. 81.01 mg g^−1^ DW in CMR35–91–63). At 12MAP, leaf total sugar in the well-watered CMR35–91–63 was recorded at 53.96 mg g^−1^ DW, which was significantly (*p* < 0.05) lower than that at 9MAP (81.01 mg g^−1^ DW), while that of the 12MAP stressed plant (70.80 mg g^−1^ DW) was reduced only slightly from the 9MAP plant (76.35 mg g^−1^ DW). Therefore, at 12MAP, the stressed plants of CMR35–91–63 had 31% higher leaf total sugar than the well-watered plants, but RY72 in both water regimes had similar leaf total sugar.

Leaf starch concentrations varied significantly (*p* < 0.05) with crop age under the well-watered conditions (Table S3), reaching maximum values at 9MAP (62.13 mg g^−1^ DW in RY72 and 71.23 mg g^−1^ DW in CMR35–91–63). Drought (30DAS and 60DAS) slightly increased starch accumulation (non-significantly) in RY72 (25% and 17% at 30 and 60DAS, respectively). In contrast, leaf starch concentration was non-significantly reduced under drought in CMR35–91–63 (−4% and −20% at 30 and 60DAS, respectively). Notably, rewatering increased starch concentration significantly (*p* < 0.05) in CMR35–91–63 (68.70 mg g^−1^ DW) compared with that at 60DAS (40.92 mg g^−1^ DW). Starch concentration in RY72 after rewatering (75.40 mg g^−1^ DW) also increased significantly from that at 60 DAS (36.68 mg g^−1^ DW). At 12MAP, starch concentrations declined significantly (*p* < 0.05) from 9MAP in both genotypes (25.92 and 27.29 mg g^−1^ DW in RY72 and CMR35–91–63, respectively), with no significant differences between water regimes.

#### 2.2.2. Changes in Reducing Sugar (RS), Non-Reducing Sugar (NRS), Total Sugar, and Starch Concentrations in Cassava Stems

**Figure 3:**
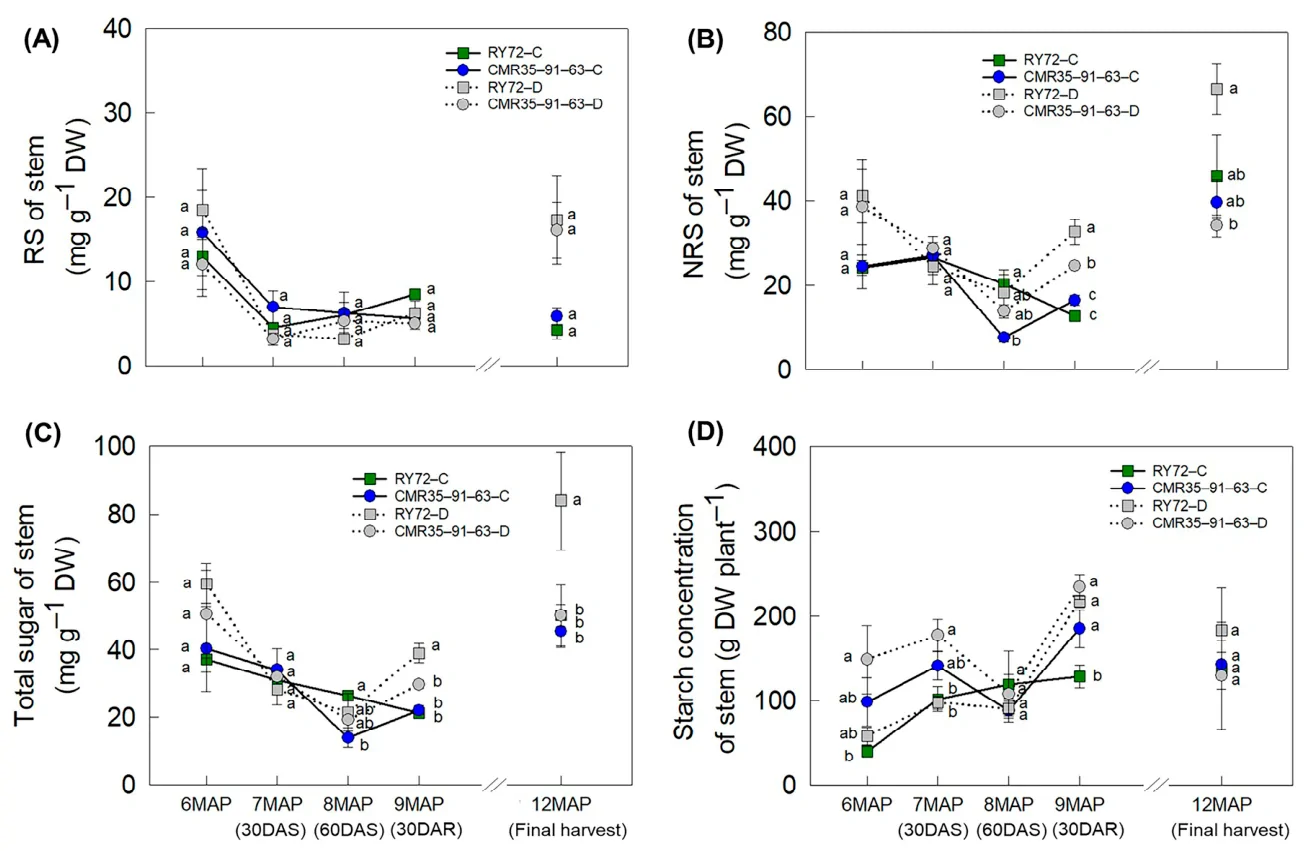
Stem reducing (**A**), non-reducing (**B**) and total sugar (**C**) and starch concentrations (**D**) of RY72 and CMR35–91–63 measured at 6, 7, 8, 9, and 12 months after planting (MAP). Plants were grown under well-watered conditions (continuous irrigation from planting to 12MAP) and drought conditions (irrigation withheld for 60 days during the dry season from 6 to 8MAP, followed by rewatering until 12MAP). Different lowercase letters indicate significant differences (*p* < 0.05 or *p* < 0.01) among water conditions and genotypes within each crop age. Data are presented as mean ± SE (n = 4).

Stem soluble carbohydrates were generally lower than those in leaves, whereas stem starch was higher than leaf starch (Figure 3; Table S4). Under the well-watered conditions, stem RS ranged from 4.29–12.97 mg g^−1^ DW in RY72 and 5.63–15.79 mg g^−1^ DW in CMR35–91–63. Drought (30DAS–60DAS) reduced stem RS by 18–47% in RY72 and 15–55% in CMR35–91–63, although these reductions were not significant. Following rewatering, stem RS remained below the control in both genotypes but increased markedly by 12MAP. In CMR35–91–63, stem RS increased significantly (*p* < 0.05) from 5.02 mg g^−1^ DW at 30 DAR (9MAP) to 16.05 mg g^−1^ DW at 12MAP, whereas RS concentration in RY72 increased significantly (*p* < 0.05) from 6.28 to 17.33 mg g^−1^ DW.

Stem NRS concentrations varied with crop age, reaching the highest values at 12MAP (45.83 and 39.59 mg g^−1^ DW in the well-watered RY72 and CMR35–91–63, respectively). Under drought (30DAS–60DAS), NRS changed little in RY72 (8–10% reduction) but increased substantially in CMR35–91–63 (82% increase at 60DAS). After rewatering, stem NRS concentrations of both genotypes were significantly (*p* < 0.05) higher than those of the 9MAP well-watered plants and were markedly higher than those of the droughted plants at 8MAP. At 12MAP, the well-watered plants showed a significant increase in stem NRS (*p* < 0.05) compared with that at 30 DAR, increasing from 12.75 to 45.83 mg g^−1^ DW in RY72 and from 16.42 to 39.59 mg g^−1^ DW in CMR35–91–63. Similarly, stem NRS in drought-stressed RY72 plants increased significantly (*p* < 0.05) from 32.65 at 9MAP to 66.57 mg g^−1^ DW at 12MAP. However, in CMR35–91–63, stem NRS at 12MAP increased slightly from the drought-stressed 9MAP.

Stem total sugar concentrations showed a pattern similar to NRS, with the highest values at 12MAP under the well-watered conditions. Drought caused only slight reductions in RY72 (9% at 30DAS and 19% at 60DAS) and CMR35–91–63 (6% at 30DAS). However, a longer drought period of 60DAS caused a 32% increase in total sugar in CMR35–91–63. Following rewatering, total sugar increased markedly in RY72 from 21.42 mg g^−1^ DW at 8MAP to 38.93 mg g^−1^ DW at 9MAP, which is significantly (*p* < 0.05) higher than that in the well-watered plant (21.27 mg g^−1^ DW). Rewatering resulted in a slight, non-significant increase in stem total sugar of CMR35–91–63 (29.62 mg g^−1^ DW) compared with the stressed plants at 8MAP (19.20 mg g^−1^ DW), and this amount was not significantly higher than that of the 9MAP well-watered plants. At 12MAP, drought-treated RY72 contained 83.91 mg g^−1^ DW of stem total sugar which was significantly (*p* < 0.05) higher than that of the well-watered plants (50.12 mg g^−1^ DW), significantly (*p* < 0.05) higher than that of the stressed plants at 9MAP (38.93 mg g^−1^ DW), and also significantly (*p* < 0.05) higher than that of the drought-stressed CMR35–91–63 (50.11 mg g^−1^ DW).

Stem starch concentrations varied significantly with crop age and genotypes. Under well-watered conditions, starch was lowest at 6MAP (39.81 mg g^−1^ DW) and continuously increased in RY72, reaching the maximum at 12MAP (135.04 mg g^−1^ DW), whereas starch in CMR35–91–63 stem fluctuated with age, showing the minimum at 8MAP (88.15 mg g^−1^ DW) and the maximum at 9MAP (185.26 mg g^−1^ DW). Drought increased stem starch in CMR35–91–63 (25% and 23% non-significant increase from the controls at 30DAS and 60 DAS, respectively). In contrast, stem starch in RY72 tended to reduce under drought (4% and 24% reduction at 30DAS and 60DAS, respectively. Rewatering caused a substantial, but not significant, increase in stem starch in both genotypes relative to that of the stressed plants at 8MAP. The 9MAP rewatered plants of RY72 accumulated the highest starch in stems (217.63 mg g^−1^ DW), significantly (*p* < 0.05) higher than the well-watered plants (128.33 mg g^−1^ DW). At 12MAP, for RY72, stem starch of the well-watered plants increased non–significantly from 128.33 mg g^−1^ DW at 9MAP to 135.04 mg g^−1^ DW, while that in the stressed plants it significantly (*p* < 0.01) reduced from 217.63 to 183.32 mg g^−1^ DW. Notably, stem starch concentration in the stressed plants of RY72 at 12MAP was markedly, although not significantly, higher than that of the CMR35–91–63 plants.

#### 2.2.3. Changes in Reducing Sugar (RS), Non-Reducing Sugar (NRS), Total Sugar, and Starch Concentrations in Cassava Tubers

**Figure 4:**
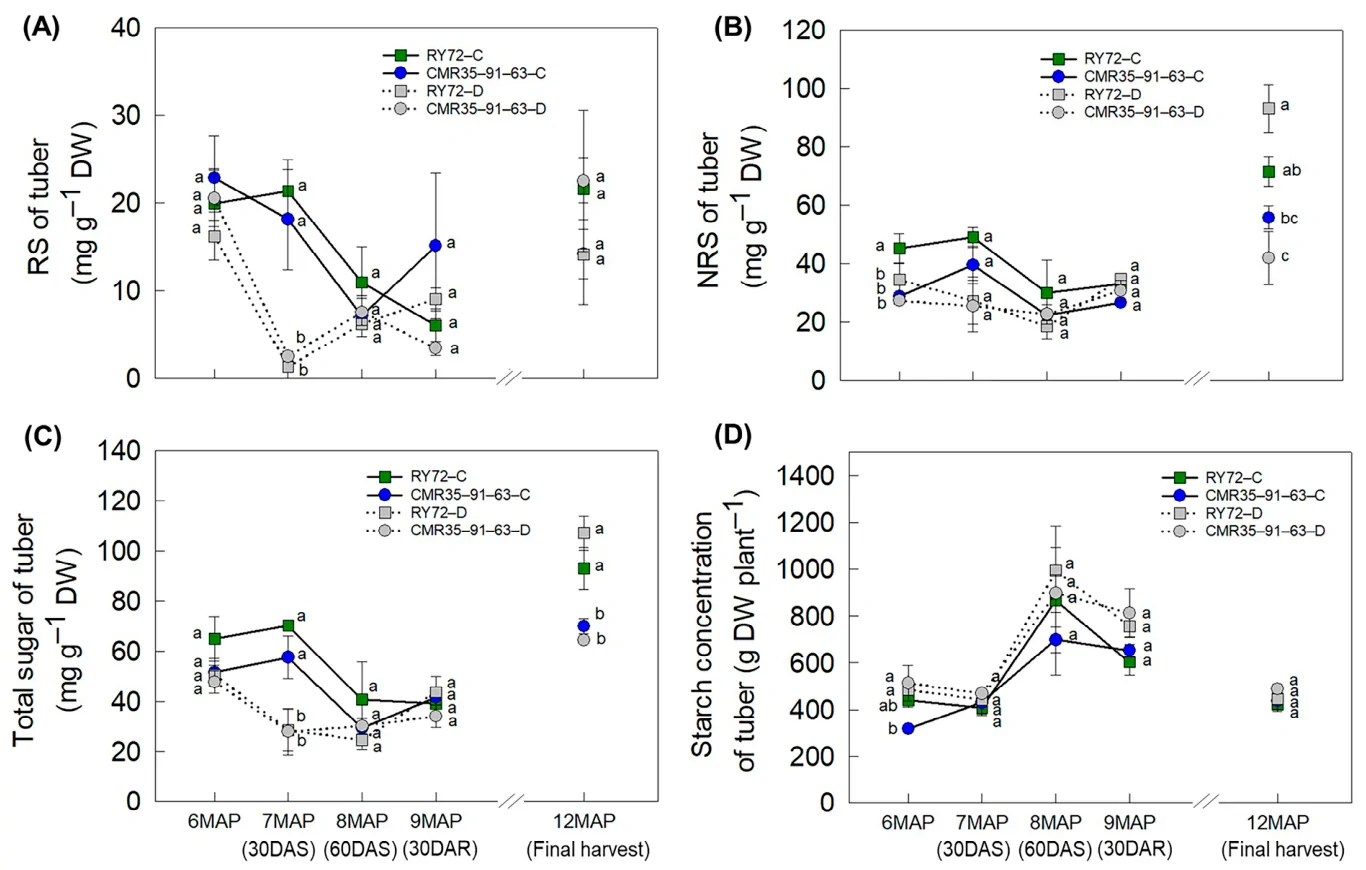
Tuber sugar (reducing (**A**), non-reducing (**B**) and total sugar (**C**)) and starch concentrations (**D**) of RY72 and CMR35–91–63 measured at 6, 7, 8, 9, and 12 months after planting (MAP). Plants were grown under well-watered conditions (continuous irrigation from planting to 12MAP) and drought conditions (irrigation withheld for 60 days during the dry season from 6 to 8MAP, followed by rewatering until 12MAP). Different lowercase letters indicate significant differences (*p* < 0.05 or *p* < 0.01) among water conditions and genotypes within each crop age. Data are presented as mean ± SE (n = 4).

Tuber soluble sugar contents (RS, NRS, and total sugar) were intermediate between those of leaves and stems, whereas tuber starch was the highest among all plant organs (Figure 4; Table S5). Under the well-watered conditions, tuber RS varied significantly with crop age in RY72 but remained relatively stable in CMR35–91–63, with the highest values at 6–7MAP (19.88–21.34 mg g^−1^ DW in RY72 and 18.13–22.85 mg g^−1^ DW in CMR35–91–63) (Figure 4A). Drought significantly (*p* < 0.05) reduced tuber RS at 30DAS by approximately 94% to 1.34 mg g^−1^ DW in RY72, and 86% to 2.49 mg g^−1^ DW in CMR35–91–63. Thereafter, RS partially recovered in RY72 during prolonged drought (6.19 mg g^−1^ DW) and after rewatering (9.02 mg g^−1^ DW), whereas recovery in CMR35–91–63 was limited until a significant (*p* < 0.05) increase at 12MAP (22.50 mg g^−1^ DW), exceeding that of RY72 (14.14 mg g^−1^ DW).

Tuber NRS was not significantly affected by water regime in either genotype, although it varied with crop age. Under drought, NRS declined by approximately 38–44% in RY72 and 36% in CMR35–91–63 compared with the controls, but these reductions were not significant. At 12MAP, drought-treated plants showed the highest NRS contents, with RY72 (93.07 mg g^−1^ DW) significantly (*p* < 0.05) exceeding CMR35–91–63 (41.83 mg g^−1^ DW) (Figure 4B).

Tuber total sugar also varied with crop age and was highest at 7 and 12MAP under the well-watered conditions. Drought significantly (*p* < 0.05) reduced tuber total sugar at 30DAS by approximately 59% in RY72 and 52% in CMR35–91–63. Prolonged drought at 60DAS slightly reduced total sugar in RY72 tuber but increased it in CMR35–91–63. Following rewatering, RY72 recovered significantly (*p* < 0.05), reaching 43.76 mg g^−1^ DW and increasing further to 107.20 mg g^−1^ DW at 12MAP, significantly higher than CMR35–91–63 (64.33 mg g^−1^ DW). In contrast, for CMR35–91–63, tuber total sugar did not increase upon rewatering at 9MAP, but it also increased significantly (*p* < 0.05) at 12MAP (Figure 4C).

Under the well-watered conditions, tuber starch concentration increased from 6 to 8 MAP, reaching its maximum at 8MAP (617.42 mg g^−1^ DW in RY72 and 697.76 mg g^−1^ DW in CMR35–91–63), before declining at 9 and 12MAP (Figure 4D). Drought slightly increased starch accumulation at 8MAP (approximately 61% relative to control in RY72 and 29% in CMR35–91–63), although the increase was generally not significant. After rewatering, tuber starch declined significantly in both genotypes (Table S5). At 12MAP, drought-treated plants exhibited the lowest starch contents (445.08 mg g^−1^ DW in RY72 and 487.23 mg g^−1^ DW in CMR35–91–63). The maximum concentration of tuber starch was, therefore, expressed in 8MAP plants (993.76 mg g^−1^ DW for RY72 and 897.37 mg g^−1^ DW for CMR35–91–63) after confronting a 60-day drought period, approximately 2.33 times (for RY72) and 2.12 times (for CMR35–91–63) more concentrated than the harvested tubers at 12MAP.

### 2.3. Malondialdehyde (MDA), Hydrogen Peroxide (H_2_O_2_) and Antioxidant Activity in Leaves of Cassava Genotypes Grown Under Drought Conditions

**Figure 5:**
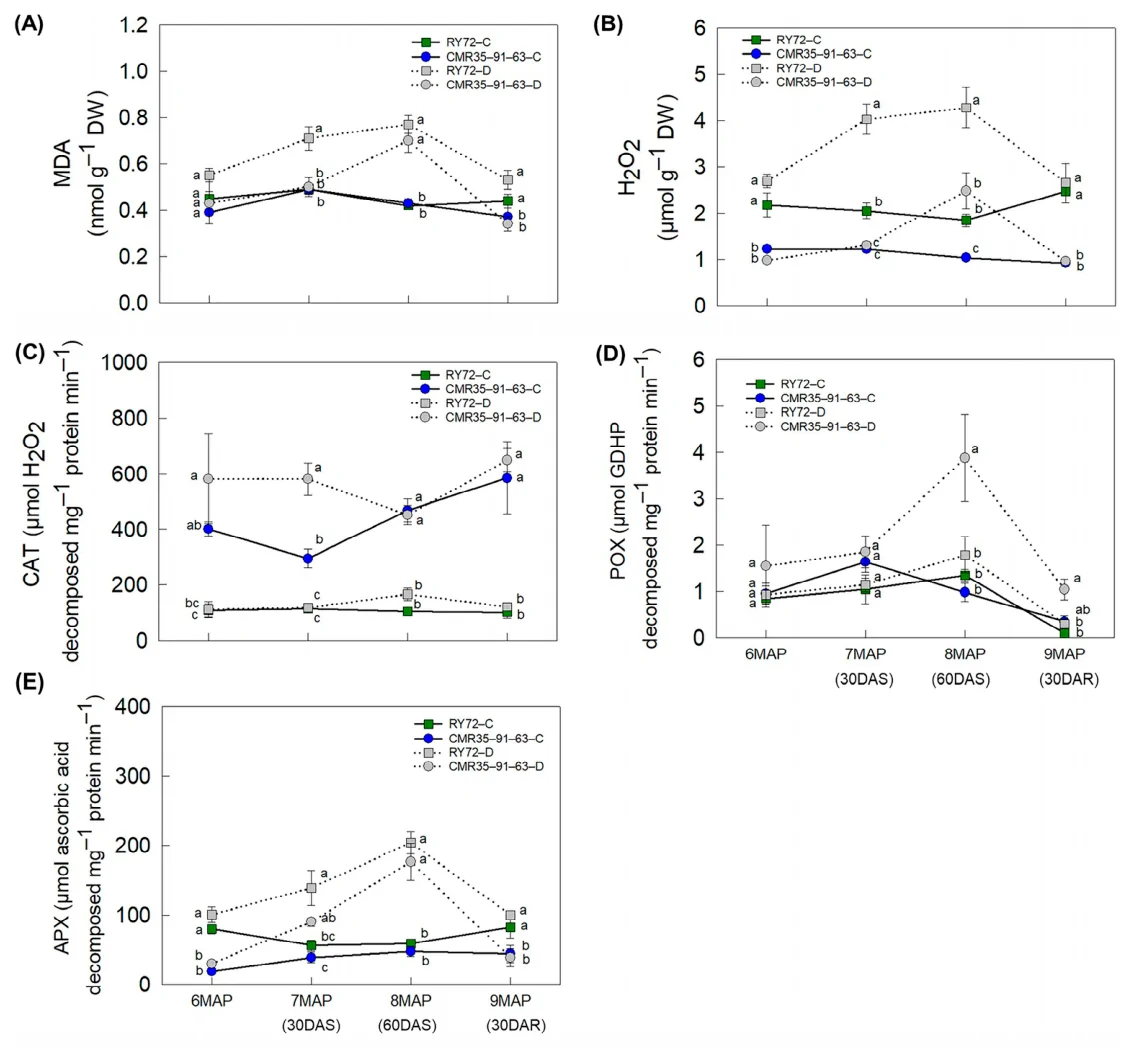
Malondialdehyde (MDA, (**A**)), hydrogen peroxide (H_2_O_2_, (**B**)), and antioxidant activity, including catalase (CAT, (**C**)), peroxidase (POX, (**D**)), and ascorbate peroxidase (APX, (**E**)) of RY72 and CMR35–91–63 measured at 6, 7, 8, and 9 months after planting (MAP). Plants were grown under well-watered conditions (continuous irrigation from planting to 12MAP) and drought conditions (irrigation withheld for 60 days during the dry season from 6 to 8MAP, followed by rewatering until 12MAP). Different lowercase letters indicate significant differences (*p* < 0.05 or *p* < 0.01) among water conditions and genotypes within each crop age. Data are presented as mean ± SE (n = 4).

Lipid peroxidation and oxidative stress increased markedly under drought (Figure 5; Table S6). Under well-watered conditions, MDA content remained low and stable across crop ages for both genotypes. At 30DAS, MDA increased significantly in RY72 (0.211 nmol g^−1^ FW; +48% relative to control), whereas the increase in CMR35–91–63 (+17%) was not significant. By 60DAS, MDA increased significantly in both genotypes to 0.244 and 0.206 nmol g^−1^ DW in RY72 and CMR35–91–63, respectively, representing significant (*p* < 0.05) increases of 77% and 69% over the controls. Following 30 days of rewatering, MDA declined significantly (*p* < 0.05) in both genotypes and returned to levels comparable with the well-watered plants.

H_2_O_2_ showed a similar response. Under the well-watered conditions, H_2_O_2_ remained stable across crop ages in RY72 (0.596–0.740 µmol g^−1^ FW), while that in CMR35–91–63 varied with age from 0.258 to 0.346 µmol g^−1^ FW. Drought significantly (*p* < 0.05) increased H_2_O_2_ in RY72 at 30DAS (+100%) and in both genotypes at 60DAS, reaching 1.358 and 0.729 µmol g^−1^ FW in RY72 and CMR35–91–63, respectively, corresponding to increases of 127% and 146% over the controls. Rewatering significantly (*p* < 0.05) reduced H_2_O_2_ in both genotypes, although RY72 maintained higher H_2_O_2_ than CMR35–91–63 throughout the experiment.

Antioxidant enzyme activities were strongly induced by drought. Under both water regimes, CAT activity was consistently higher in CMR35–91–63 than in RY72. At 30DAS, CAT activity increased by 126% in CMR35–91–63, but remained stable in RY72. With prolonged drought (60DAS), CAT activity of RY72 significantly (*p* < 0.05) increased by 176% over the control, and that of CMR35–91–63 significantly (*p* < 0.05) increased by 61%. After rewatering, CAT activity in both genotypes significantly (*p* < 0.05) declined compared to that at 8MAP.

POX activity was relatively stable under well-watered conditions for RY72 from 6 to 8MAP but significantly reduced at 9MAP, whereas that of CMR35–91–63 fluctuated from the maximum at 7MAP to the minimum at 9MAP. Under drought (30DAS), POX activity showed slight, non-significant increases. Notably, at 60DAS, POX activity increased significantly (*p* < 0.05) by approximately 537% in CMR35–91–63. Following rewatering, POX activity declined significantly (*p* < 0.05) in both genotypes, with CMR35–91–63 maintaining higher activity than RY72. In contrast to CAT, APX activity was consistently higher in RY72 than in CMR35–91–63 under well-watered conditions. Drought significantly (*p* < 0.05) enhanced APX activity in both genotypes at 30DAS (+150% in RY72 and +162% in CMR35–91–63), with further increases at 60DAS to 240% (for RY72) and 286% (for CMR35–91–63) over the controls, respectively. After rewatering, APX activity decreased significantly (*p* < 0.05) and returned to levels not significantly different from those of the well-watered plants.

### 2.4. Growth and Yield of Cassava 6 to 9 Months and Final Harvest (12 Months) Under Different Water Treatments

**Figure 6:**
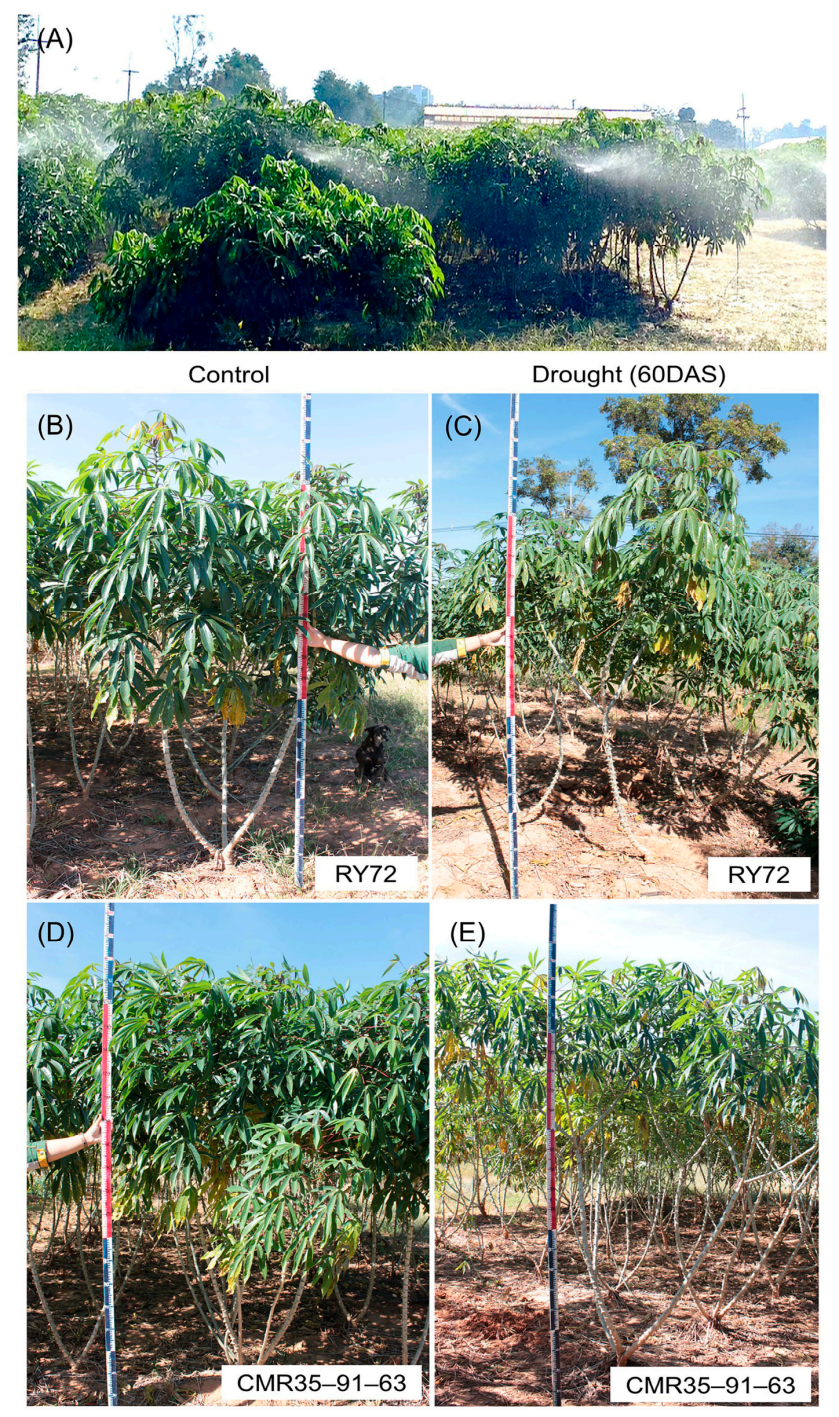
Morphology of cassava plants at 6 months after planting (MAP). Plants were irrigated using a mini-overhead sprinkler irrigation system (**A**). Representative plants of the cassava genotypes RY72 (**B**,**D**) and CMR35–91–63 (**C**,**E**) grown under well-watered and drought conditions after 60 days of drought stress (60 DAS) are shown. The experiment was conducted at the Field Crops Research Station, Faculty of Agriculture, Khon Kaen University, Khon Kaen, Thailand.

**Figure 7:**
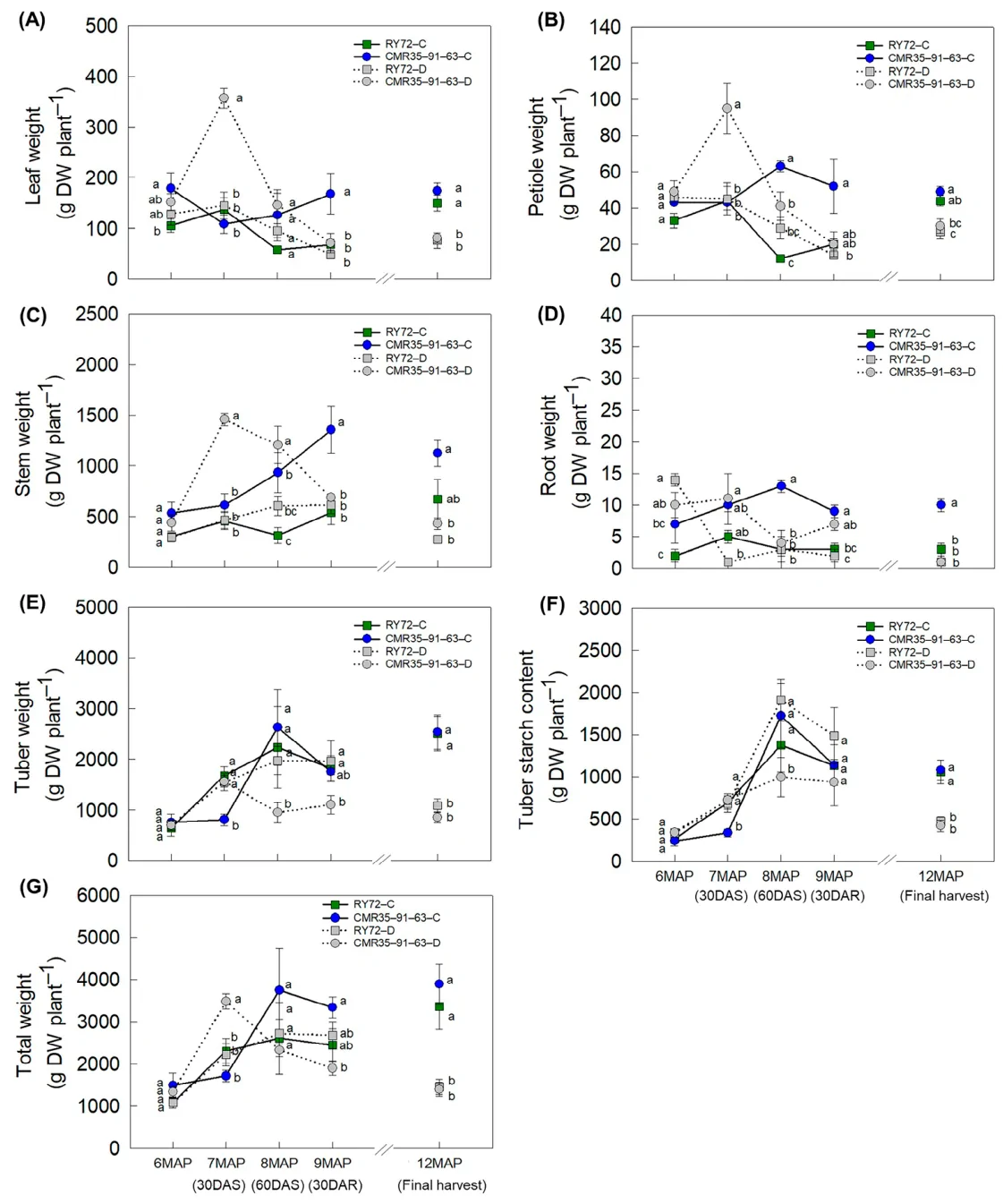
Leaf dry weight (DW, (**A**)), petiole DW (**B**), stem DW (**C**), root DW (**D**), tuber DW (**E**), tuber starch content (**F**), and total dry weight (DW, (**G**)) of RY72 and CMR35–91–63 measured at 6, 7, 8, 9, and 12 months after planting (MAP). Plants were grown under well-watered conditions (continuous irrigation from planting to 12MAP) and drought conditions (irrigation withheld for 60 days during the dry season from 6 to 8MAP, followed by rewatering until 12MAP). Different lowercase letters indicate significant differences (*p* < 0.05 or *p* < 0.01) among water conditions and genotypes within each crop age. Data are presented as mean ± SE (n = 4).

Growth and morphology of the two cassava genotypes under well-watered and drought conditions are shown in Figure 6. Dry weight (DW) of leaves, petioles, stems, roots, tubers, total biomass and tuber starch content were measured at 6, 7, 8, 9, and 12MAP (Figure 7; Table S7). Under the well-watered conditions, leaf DW of RY72 was highest at 6–7MAP and declined significantly (*p* < 0.05) at 8MAP before partially recovering at 9–12MAP, while leaf DW of CMR35–91–63 remained relatively stable from 6 to 12MAP. In contrast, stem and tuber DW increased progressively with crop age, reaching maximum values at 12MAP, while root DW remained relatively stable. At the final harvest (12MAP), CMR35–91–63 produced markedly greater stem DW (1124 g plant^−1^) than RY72 (670 g plant^−1^) and slightly higher tuber DW (2537 vs. 2506 g plant^−1^). Regarding tuber starch content, well-watered plants showed the highest values at 8MAP (1375 and 1726 g plant^−1^ for RY72 and CMR35–91–63, respectively), whereas tuber starch content at 9 and 12MAP was significantly lower (*p* < 0.05) than that at 8MAP, reaching approximately 1139 and 949 g plant^−1^ for RY72 and 1143 and 1083 g plant^−1^ for CMR35–91–63, respectively (Figure 7F).

Short–term drought (30DAS) did not reduce plant biomass and, in several organs, promoted biomass accumulation, particularly in CMR35–91–63. Compared with the well-watered control, leaf, petiole, stem, tuber, and total DW of CMR35–91–63 increased by approximately 232%, 120%, 139%, 94%, and 103%, respectively, whereas RY72 showed no significant increases (leaf, petiole and stem) or decreases (root, tuber and total). After 30DAS, tuber starch content of CMR35–91–63 showed a significant (*p* < 0.05) increase of approximately 117% compared with the well-watered control, reaching 729 g plant^−1^, whereas RY72 showed no significant difference from the control. Under prolonged drought (60DAS), CMR35–91–63 showed a significant (*p* < 0.05) reduction in DW of petiole, root and tuber (−35%, −70% and –64%, respectively), while leaf and stem DW showed non-significant increases of 16% and 29%, respectively. On the other hand, RY72 subjected to 60DAS exhibited 64%, 144% and 95% (non-significant) increases in leaf, petiole and stem DW, respectively, and 9% and 32% reductions in root and tuber DW. Tuber starch content of RY72 increased by 39% compared with the control, reaching 1913 g plant^−1^, whereas that of CMR35–91–63 significantly (*p* < 0.05) decreased by 51%, down to 851 g plant^−1^.

Following 30 days of rewatering (30DAR), biomass recovery differed between genotypes. CMR35–91–63 exhibited significant (*p* < 0.05) reductions in leaf DW (−58%) and stem DW (−42%) relative to the well-watered control, whereas RY72 showed only minor changes and maintained comparable biomass of all organs between the two water regimes. Upon rewatering, tuber DW was reduced by 37% (non-significant) in CMR35–91–63 but increased by 8% (non-significant) in RY72. Tuber starch content of RY72 increased by 31% (non-significant) compared with the control, whereas that of CMR35–91–63 decreased by 18% (non–significant).

At the final harvest (12MAP), drought significantly reduced biomass and starch content in both genotypes. Leaf, petiole, stem, root, tuber, and total DW remained lower than in the well-watered plants, with greater reductions in CMR35–91–63 than in RY72. Tuber DW decreased by approximately 52% in RY72 and 66% in CMR35–91–63, while total DW declined by 57% and 64%, respectively. In response to drought, tuber starch content declined by 55% from 1060 to 474 g plant^−1^ in RY72, and 61% from 1083 to 421 g plant^−1^ in CMR35–91–63. These results suggested that RY72 maintained biomass production better than CMR35–91–63 under prolonged drought.

### 2.5. Principal Component Analysis (PCA) and Correlations Among Physiological Parameters, Growth, and Yield of Cassava

**Figure 8:**
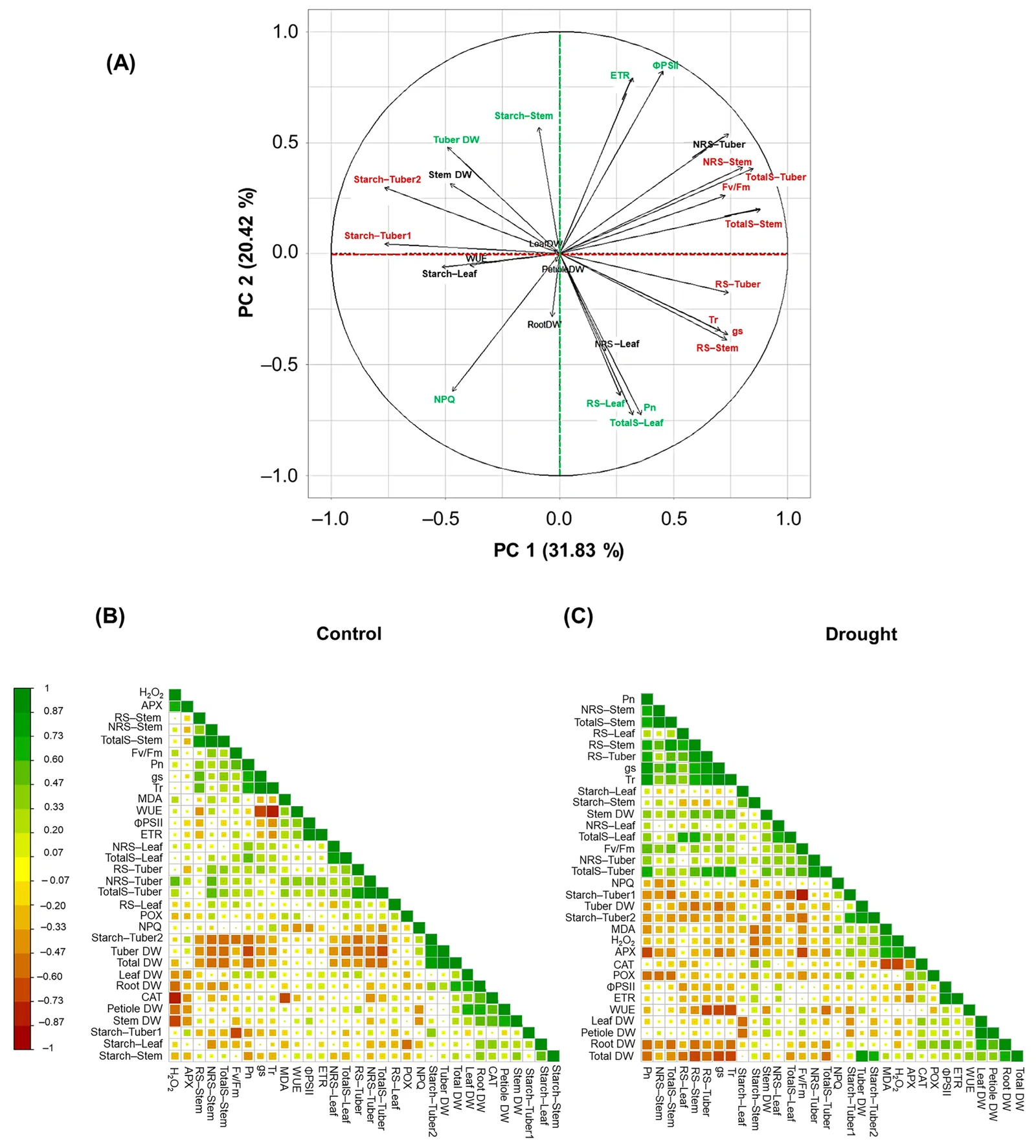
Principal component analysis (PCA, (**A**)) and correlation matrix of photosynthetic parameters (Fv/Fm, ΦPSII, ETR, NPQ, Pn, gs, Tr, and WUE), carbohydrate contents (leaf, stem, and tuber reducing sugars (RS), non-reducing sugars (NRS), total sugars, and starch), plant dry weight (DW; leaves, petioles, stems, roots, and tubers), and tuber yield (tuber DW) of the cassava genotypes RY72 and CMR35–91–63 at 6, 7, 8, 9, and 12 months after planting (MAP) under well-watered (control, (**B**)) and drought conditions (irrigation withheld for 60 days from 6 to 8 MAP, (**C**)). The red fonts in the loading plot (**A**) are the parameters mainly associated with PC1, and the green fonts with PC2.

The PCA of photosynthetic traits (Fv/Fm, ΦPSII, ETR, NPQ, Pn, gs, Tr, and WUE), carbohydrate contents (RS, NRS, and total sugars in leaves, stems, and tubers), and dry biomass traits of RY72 and CMR35–91–63 under different water regimes is presented in Figure 8A. The first two principal components explained 52.25% of the total variance, with PC1 and PC2 accounting for 31.83% and 20.42%, respectively. PC1 was mainly associated with carbohydrate accumulation in stems and tubers, photosynthetic performance (Fv/Fm, gs, and Tr), tuber starch concentration, and tuber starch content at final harvest. In contrast, PC2 was primarily associated with ΦPSII, ETR, NPQ, Pn, tuber dry weight (DW) at final harvest, leaf RS, leaf total sugar, and stem starch, indicating variation in plant growth and photosynthetic performance.

Pearson’s correlation analysis revealed distinct relationships among physiological traits, carbohydrate metabolism, antioxidant activity, and dry biomass under well-watered and drought conditions (Figure 8B,C). Under well-watered conditions, tuber DW was positively correlated (*p* < 0.05) with tuber starch content (*r* = 0.86) and total DW (*r* = 0.93) but negatively correlated with Pn (*r* = −0.61), gs (*r* = −0.43), leaf total sugar (*r* = −0.47), stem RS, NRS and total sugar (*r* = −0.41, −0.50 and −0.53, respectively), tuber RS, NRS and total sugar (*r* = −0.53, −0.50 and −0.61, respectively). Tuber starch concentration was positively correlated (*p* < 0.05) only with tuber starch content (*r* = 0.44) but was negatively correlated with Fv/Fm (*r* = −0.65) and Pn (*r* = −0.37) (Figure 8B).

Under drought conditions, tuber DW remained positively correlated (*p* < 0.05) with tuber starch content (*r* = 0.82), total DW (*r* = 0.79), and H_2_O_2_ (*r* = 0.43), but was negatively correlated with gs (*r* = −0.49), Tr (*r* = −0.45), leaf RS (*r* = −0.42), stem RS (*r* = −0.48), tuber RS (r = −0.39) and stem DW (*r* = −0.41). Tuber starch concentration was negatively correlated with Fv/Fm (*r* = −0.75), Pn (*r* = −0.46), WUE (*r* = −0.38), leaf NRS and total sugar (*r* = −0.43 and −0.48, respectively), stem NRS and total sugar (r = −0.45 and −0.47, respectively), but positively correlated with tuber starch content (*r* = 0.66), POX (*r* = 0.35), and APX (*r* = 0.42) (Figure 8C). Among the antioxidant enzymes, CAT was strongly negatively correlated with H_2_O_2_ (*r* = −0.71) and MDA (*r* = −0.67), whereas APX was strongly positively correlated (*p* < 0.01) with H_2_O_2_ (*r* = 0.72) and MDA (*r* = 0.69), indicating a close association between oxidative stress markers and antioxidant enzyme responses under drought conditions.

## 3. Discussion

### 3.1. The Effect of Water Stress on Photosynthetic Performance of Cassava at Late Growth Stage (5 to 8 Months After Planting, MAP)

The present study demonstrated that cassava RY72 and CMR35–91–63 possess a high degree of photosynthetic resilience to prolonged drought (0 to 60 days without irrigation and complete lack of rainfall) during the late (tuber-bulking) growth stage (6–8MAP), as shown by the non-significant reductions in Fv/Fm, ΦPSII, ETR and Pn relative to control (Figure 1 and Supplementary Tables S1 and S2). Compared with a previous study [15], photosynthetic performance of the younger plants (3–6MAP) of RY72 and CMR35–91–63 under prolonged drought (60DAS) was more strongly affected than the older plants in this study. Despite exposure to 30 and 60 days of water deficit, neither chlorophyll fluorescence nor gas exchange parameters were significantly impaired, suggesting that the imposed drought was insufficient to cause severe damage to the photosynthetic machinery. The stability of Fv/Fm under drought indicated that Photosystem II (PSII) remained structurally intact and was not subjected to substantial photoinhibition [31,32]. The Fv/Fm values remained close to the optimal range (approximately 0.78–0.83), supporting previous reports that cassava is capable of maintaining PSII integrity under moderate drought through efficient photoprotective mechanisms [33,34]. Instead, the decline in Fv/Fm with increasing crop age suggests that developmental senescence rather than drought primarily influenced PSII efficiency. Similarly, the relatively constant ΦPSII and ETR demonstrated that the efficiency of photochemical energy conversion and electron transport was largely maintained throughout the drought period [35,36]. These results suggested that the light reactions of photosynthesis remained relatively well maintained, supporting continued photochemical energy conversion and electron transport under limited water availability [32]. The slight reduction in ΦPSII observed only in drought-treated CMR35–91–63 at 12MAP indicates that this genotype experienced greater sensitivity during late plant development. The tendency for higher NPQ in CMR35–91–63 under drought suggested an activation of photoprotective heat dissipation to prevent excess excitation energy from damaging PSII [37,38]. The increase in NPQ likely reflected an enhanced photoprotective response, whereby excess excitation energy was dissipated as heat when photosynthetic energy utilization was constrained under drought conditions [38,39]. In contrast, the relatively stable NPQ in RY72 implies that this genotype experienced less excitation pressure, consistent with its superior maintenance of photochemical efficiency. Gas exchange measurements further supported the fluorescence results, except at the mature stage (12MAP). Although Pn, g_s_, and Tr declined numerically under drought, the absence of significant reductions indicates that stomatal limitation remained relatively mild during tuber bulking [21]. The larger reductions observed in CMR35–91–63 suggested greater stomatal sensitivity to water deficit, which likely contributed to its lower photosynthetic performance. Nevertheless, the rapid recovery of Pn following rewatering demonstrated that the reductions were largely reversible and reflected temporary stomatal regulation rather than irreversible metabolic damage [27,40]. Interestingly, Pn declined with plant age despite partial recovery of gs, Fv/Fm, φPSII and ETR at 12MAP, indicating that PSII photochemical function remained relatively well maintained in mature cassava plants, whereas CO_2_ assimilation was constrained downstream of PSII. The lower Pn may reflect limitations in biochemical CO_2_ fixation, mesophyll CO_2_ diffusion, potentially associated with age-related declines in photosynthetic enzyme activity during late development. Although WUE varied significantly among developmental stages, drought itself produced relatively small changes. This suggested that developmental processes exerted a stronger influence on water use efficiency than soil water availability during the tuber bulking stage [41]. The decline in WUE at 60DAS and at final harvest may reflect age-related reductions in photosynthetic capacity rather than changes in transpiration alone. Overall, RY72 exhibited greater drought tolerance than CMR35–91–63. It maintained higher ΦPSII, Pn, gs, and Tr under drought, showed smaller reductions in photosynthetic performance, and required less photoprotective energy dissipation (NPQ). These characteristics indicated that RY72 possesses superior physiological mechanisms for maintaining carbon assimilation under water deficit, making it better adapted to drought during the tuber bulking stage.

### 3.2. The Effect of Water Stress on Carbohydrate Partitioning of Cassava at Tuber Bulking-Growth Stage

Drought had only limited effects on source leaves but substantially altered sink carbohydrate allocation. The relatively stable concentrations of soluble sugars and starch in leaves indicated that leaf carbon metabolism was maintained during drought, consistent with the minimal reductions observed in photosynthetic parameters [13,42]. Since photosynthesis remained largely unaffected, leaves likely continued supplying assimilates despite reduced soil moisture. This agrees with the chlorophyll fluorescence and gas exchange results, which showed little impairment of PSII function or CO_2_ assimilation during the tuber bulking stage. The slight accumulation of leaf starch during drought suggests that temporary reductions in assimilate export may have occurred, allowing excess photosynthates to be stored transiently within leaves [23,43]. However, because these increases were not statistically significant, leaf carbohydrate metabolism appears to be relatively drought-tolerant.

Stems acted as temporary carbohydrate reservoirs during drought [5,42,44]. One of the most striking findings was the marked increase in stem NRS, total sugars, and starch, particularly during prolonged drought. Cassava stems are well recognized as important storage organs that buffer fluctuations in carbon supply and demand [45,46]. Under drought, assimilates that would normally be transported to developing storage roots may instead accumulate in stems because root sink activity declines. The strong accumulation of stem carbohydrates therefore suggested that drought restricted carbon translocation to storage roots more than carbon fixation itself. This interpretation is consistent with previous studies showing that cassava temporarily stores carbohydrates in stems during stress before remobilizing them after favorable conditions return [13,24].

Contrasting carbon allocation strategies between genotypes was clearly evident. Although both genotypes maintained photosynthesis, they partitioned carbohydrates differently under drought. RY72 showed relatively stable stem carbohydrates, faster recovery of tuber reducing sugar, rapid restoration of tuber total sugar after rewatering, and higher soluble sugar concentrations in tubers at final harvest. These responses suggested an efficient remobilization of stored carbohydrates from vegetative tissues toward storage roots following drought relief. Such flexibility in source–sink relationships is characteristic of drought-tolerant cassava genotypes [13,46,47]. In contrast, CMR35–91–63 accumulated considerably larger amounts of stem sugars and starch during drought while showing slower recovery of tuber sugars. This pattern suggested that carbohydrates remained stored in stems for longer periods, reflecting reduced sink strength or less efficient assimilate transport to storage roots. Although CMR35–91–63 eventually retained higher tuber starch at final harvest, its lower soluble sugar recovery indicates delayed carbon remobilization.

Drought preferentially affected soluble sugars rather than starch. The strongest drought effects occurred in tuber reducing sugar and total sugar, whereas starch was comparatively stable or even increased during moderate drought. Reducing sugars represent metabolically active carbohydrates that respond rapidly to changes in source–sink balance in cassava [16]. Their sharp decline immediately after drought suggests rapid utilization for osmotic adjustment and maintenance metabolism or reduced phloem import into storage roots. In contrast, starch accumulation continued during moderate drought, particularly at 8MAP, implying that cassava maintained storage root growth despite limited water availability. Only during prolonged stress and at final harvest did starch decline substantially, indicating that long-term drought eventually reduced carbon deposition into storage roots.

Developmental stage exerted a stronger influence than drought. Many carbohydrate traits changed significantly with crop age irrespective of water treatment. Examples include: declining leaf reducing sugars, increasing stem carbohydrate reserves, peak tuber starch accumulation at 8MAP, and declining starch toward physiological maturity. These observations indicate that developmental regulation had a greater influence on carbohydrate partitioning than moderate drought imposed during tuber bulking [5,22,44]. Therefore, crop age should be considered an important factor when interpreting drought responses in cassava. The carbohydrate data strongly support the physiological measurements presented earlier. Despite prolonged drought, Fv/Fm remained stable, ΦPSII and ETR were largely maintained, Pn showed only moderate reductions, and gs was only slightly affected. Because carbon assimilation remained relatively stable, the major drought response occurred after photosynthesis, through changes in assimilate partitioning among organs rather than reduced carbon fixation [23,27]. This indicates that cassava drought tolerance during tuber bulking depends primarily on maintaining photosynthesis while adjusting sink allocation and carbohydrate storage.

### 3.3. The Effect of Water Stress on MDA, H_2_O_2_ and Antioxidant Activity of Cassava

Drought-induced oxidative stress but not irreversible oxidative damage [28,48]. Both MDA and H_2_O_2_ increased progressively during drought, demonstrating that water deficit enhanced reactive oxygen species (ROS) production and lipid peroxidation [26]. Increased MDA is a widely accepted indicator of membrane oxidative damage and lipid peroxidation, commonly assessed via the TBARS method [49], while H_2_O_2_ reflects ROS accumulation associated with environmental stress and redox signaling [50]. However, despite these increases, absolute MDA values remained relatively low and rapidly returned to control levels after rewatering. Together with the recovery of photosynthesis observed previously, these findings indicate that drought caused transient oxidative stress rather than permanent cellular injury [51,52]. Increased ROS may function as signaling molecules that regulate stress acclimation pathways rather than only causing damage [48].

An interesting observation is that RY72 consistently maintained higher H_2_O_2_ concentrations than CMR35–91–63, even under well-watered conditions. Higher H_2_O_2_ is not necessarily detrimental, as moderate concentrations are increasingly recognized as signaling molecules that activate drought-responsive pathways, antioxidant enzymes, osmotic adjustment, and stress-related gene expression [26,50]. Therefore, the elevated H_2_O_2_ in RY72 may represent a stronger ROS signaling capacity rather than greater oxidative damage, particularly because MDA levels remained comparable between genotypes and photosynthesis was maintained throughout drought.

Antioxidant enzymes were strongly induced during prolonged drought [53]. The progressive increases in CAT, POX, and APX demonstrate that plants activate an efficient enzymatic antioxidant system under water deficit [54]. Among these enzymes, APX exhibited the strongest induction in both genotypes. CAT showed substantial increases, particularly during prolonged drought, while POX responded later, especially in CMR35–91–63. This sequential activation may reflect coordinated detoxification of ROS as drought severity increased [28]. APX appears to play a dominant role in H_2_O_2_ detoxification. The most striking response was the large induction of APX. Because APX has a higher affinity for H_2_O_2_ than CAT, it is particularly effective in scavenging H_2_O_2_ at relatively low concentrations and plays an important role in maintaining H_2_O_2_ homeostasis in chloroplasts, cytosol, and other cellular compartments [55,56]. The strong APX response likely contributed substantially to maintaining chloroplast integrity and protecting PSII, consistent with the stable Fv/Fm, ΦPSII, and ETR observed in the photosynthetic analysis.

Genotypes employed different antioxidant strategies. Although both genotypes recovered successfully after rewatering, their antioxidant responses differed, consistent with genotype-dependent redox regulation [57]. RY72 exhibited higher basal H_2_O_2_, higher basal APX, moderate CAT activity, and relatively small POX induction. These observations suggest that RY72 may rely on rapid ROS signaling coupled with efficient APX-mediated detoxification, allowing oxidative stress to be controlled before extensive membrane damage occurs. CMR35–91–63 exhibited lower basal H_2_O_2_, much higher CAT activity, and very strong POX induction during prolonged drought, indicating a strategy based on greater antioxidant enzyme capacity to remove accumulated H_2_O_2_ [54]. Oxidative stress responses were consistent with photosynthetic performance. Although drought increased ROS production, PSII efficiency remained stable and electron transport was maintained, indicating effective photoprotection [50]. These findings suggest that antioxidant activation effectively prevented oxidative damage to the photosynthetic apparatus. The rapid decline of MDA, H_2_O_2_, CAT, POX, and APX following rewatering further suggests that plants possess a highly resilient antioxidant system capable of restoring cellular redox homeostasis after drought relief [57,58]. Combining all physiological results, photosynthesis remained largely functional with minimal reductions in photochemical efficiency, ROS production increased under drought but was effectively regulated, antioxidant enzymes (especially APX and CAT) were strongly induced, limiting oxidative injury, and rapid recovery after rewatering indicates reversible drought effects rather than permanent cellular damage [26,48,51].

### 3.4. The Effect of Water Stress on Growth and Yield of Cassava at Late Growth Stage

Drought had relatively small short-term effects but substantial long-term impacts on biomass production [59]. The physiological measurements demonstrated that drought may have contributed to only modest reductions in photosynthesis, whereas biomass data reveal that prolonged drought eventually translated into substantial reductions in growth and yield. This apparent discrepancy suggests that maintenance of photosynthetic capacity alone was insufficient to sustain biomass accumulation during extended drought [60,61]. Instead, reduced assimilate utilization, altered carbon partitioning, and slower organ expansion likely became increasingly important as drought progressed [11,62]. Early biomass increases likely reflect continued growth before drought fully affected sink activity.

One notable observation was the significant increases in leaf, stem, and tuber biomass during the first 30 days of drought, particularly in CMR35–91–63. Although Pn and gs under drought at 30 DAS were lower than those of the well-watered control, with Pn decreasing by approximately 23%, CMR35–91–63 under drought developed a substantially greater number of leaves, resulting in a 232% greater leaf area compared with the control. Thus, although photosynthetic rate per unit leaf area was reduced under drought, the substantially greater photosynthetic surface may have contributed to maintaining assimilate production and biomass accumulation during the early drought period. Several mechanisms may explain this response. First, drought was imposed during the tuber bulking stage, when plants had already established substantial canopy and storage roots. Existing assimilates may therefore have continued supporting growth despite declining soil moisture [24]. Second, carbohydrate results showed increased starch accumulation in stems and storage roots during early drought, suggesting that assimilate production temporarily exceeded sink limitation. Thus, early biomass increases probably represent continued utilization of pre-existing carbohydrate reserves rather than stimulation of growth by drought itself. RY72 maintained growth more effectively during prolonged drought. Although both genotypes experienced reductions in biomass, RY72 consistently showed smaller declines. Compared with CMR35–91–63, RY72 exhibited smaller reductions in leaf biomass, greater stem stability, less reduction in total biomass, and higher tuber biomass at harvest. These findings are in close agreement with previous growth and yield results, which showed that RY72 exposed to early drought stress (4–5 MAP) maintained more efficient carbohydrate remobilization and exhibited faster recovery after rewatering [15]. Therefore, the growth performance of RY72 appears to result from cumulative advantages in physiological resilience throughout the drought period.

**Figure 9:**
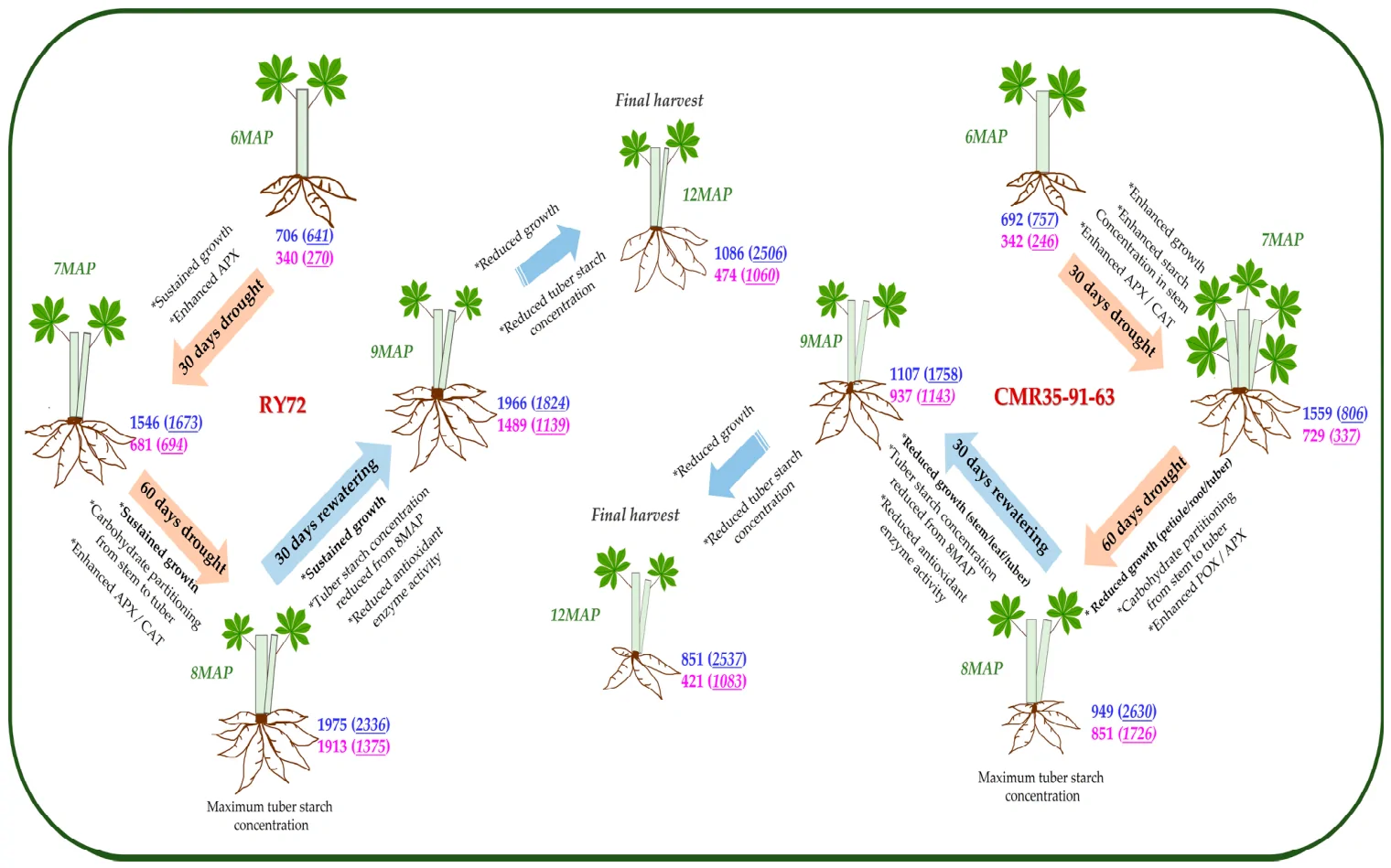
Schematic diagram showing growth and physiological changes in cassava cvs. RY72 and CMR35–91–63 subjected to drought stress for 60 days during late growth stage from 6MAP to 8MAP, rewatered for 30 days (9MAP), then allowed to grow under irrigation until final harvest (12MAP). The mean tuber dry weight (g) and tuber starch content (g plant^−1^) of the drought-treated plants are indicated by blue and pink values, respectively. The underlined blue and pink values in the brackets represent the mean tuber dry weight (g) and tuber starch content (g plant^−1^) of the continuously well-watered plants. (MAP, months after planting; APX, ascorbate peroxidase; CAT, catalase; POX, peroxidase). The asterisk (*) indicates a significant change in growth, physiological, and biochemical responses in each cassava genotype.

Carbohydrate partitioning explains higher tuber yield in RY72 than in CMR35–91–63. The biomass results strongly support the carbohydrate partitioning data. During drought, for RY72, photoassimilates were predominantly partitioned to tuber, resulting in very high tuber DW at 8MAP but relatively low above-ground DW. In contrast, CMR35–91–63 partitioned more photoassimilates to support stem and leaf growth rather than tuber (Figure 7; Table S7). At 8MAP, even though both genotypes had similar tuber DW in the well-watered condition, under stress, RY72 exhibited two-fold greater tuber DW than CMR35–91–63. Similar to CMR35–91–63, previous research found that in several crops, drought-induced sink limitation and altered phloem transport activity, leading to less partitioning to sink organs like storage roots [62,63]. After rewatering, biomass lost during prolonged drought could not be completely compensated after rewatering, suggesting that reduced carbon assimilation and dry matter accumulation during storage root bulking imposed a lasting constraint on final yield [27,64,65]. In this study, final tuber DW of the stressed plants of both genotypes was significantly reduced compared with the controls, with RY72 showing a lower percentage reduction associated with higher Pn and sink activity than CMR35–91–63, resulting in higher tuber DW and starch content at the final harvest (12MAP). Taken together, as outlined in Figure 9, RY72 consistently outperformed CMR35–91–63 throughout these processes by maintaining higher photosynthetic activity, greater photochemical stability, more efficient assimilate partitioning to tubers, better recovery after rewatering, and higher tuber and starch yield.

## 4. Materials and Methods

### 4.1. Experimental Site and Environmental Conditions

The field experiment was conducted at the Field Crops Research Station, Faculty of Agriculture, Khon Kaen University, Khon Kaen, Thailand (16°28′29.7″ N, 102°48′37.3″ E; 195 m above sea level). The study area is characterized by a tropical savanna climate (Aw) according to the Köppen climate classification system [66], which is common in many regions of Africa, South America, and Asia. The climate is characterized by distinct wet and dry periods, with drought conditions frequently occurring during the dry season. In Thailand, seasonal variation is primarily influenced by monsoonal circulation and can be classified into three distinct seasons: the hot season (March–May), the rainy season or southwest monsoon (June–October), and the cool season or northeast monsoon (November–February). Meteorological variables, including light intensity, air temperature (T_air_), and total rainfall, were recorded at 24-hour intervals from planting until harvest (May 2022–May 2023) using an automated weather station (WatchDog 2000, Spectrum Technologies Inc., Lincoln, NE, USA).

Weather conditions: the mean light intensity during the rainy, cool, and hot seasons was 479, 474, and 520 µmol photon m^−2^ s^−1^, respectively (Table 1). The corresponding mean air temperatures were 26.3, 23.7, and 30.2 °C, with average minimum temperatures of 23.3, 17.7, and 24.7 °C and average maximum temperatures of 29.3, 29.6, and 35.6 °C, respectively (Table 1). Total rainfall during the rainy season reached 1339 mm across 135 rainy days, whereas rainfall during the cool and hot seasons was 77 mm (26 rainy days) and 363 mm (74 rainy days), respectively.

Soil characteristics: the experimental field is classified as a Yasothon soil series (fine-loamy, siliceous, isohyperthermic, Oxic Paleustults), one of the dominant soil groups in northeastern Thailand and a soil type considered suitable for cassava cultivation. Soil samples were collected before planting at depths of 0–30 cm and 30–60 cm to determine physical and chemical properties. Soil texture was analyzed using the hydrometer method, whereas soil organic matter was determined following the Walkley-Black procedure [67]. Soil pH, total nitrogen (Kjeldahl method), available phosphorus (Bray II method), exchangeable potassium (1 M ammonium acetate extraction, pH 7.0), electrical conductivity (EC; 1:5 soil-to-water ratio), and cation exchange capacity (CEC; 1 M ammonium acetate extraction, pH 7.0) were analyzed according to the methods described by Baker and Norman [68]. The soil exhibited a sandy loam texture, comprising more than 73% sand, 19.26–21.08% silt, and 5.19–7.30% clay. Across the 0–60 cm soil profile, available phosphorus (P) ranged from 18.75 to 28.13 mg kg^−1^, exchangeable potassium (K) from 23.94 to 27.00 mg kg^−1^, total nitrogen (N) from 0.032% to 0.034%, organic matter from 0.55% to 0.58%, and cation exchange capacity from 2.5 to 3.2 cmol kg^−1^ (Table 2).

Soil moisture content in both fully irrigated and drought-treated plots was determined using the gravimetric method at the soil depths of 0–30 and 30–60 cm at the crop ages 6, 7, 8, 9 and 12MAP. Field capacity and permanent wilting point were estimated using a pressure plate apparatus [69]. Before drought (6MAP; 0 days after stress, DAS), soil moisture content at a depth of 0–60 cm showed a non-significant difference between the irrigated and drought-treatment plots (12.12–12.50%) (Table S2). However, at 7 and 8MAP (30–60 DAS), soil moisture content in the drought plots (3.98–8.65%) significantly (*p* < 0.05) decreased compared with the control plots (9.70–13.15%). Following rewatering, soil moisture content in the drought treatment (6.49–11.26%) recovered to levels comparable to those of the control (7.03–11.42%). By the final harvest (12MAP), soil moisture was not significantly different between the water treatments (Table 2).

### 4.2. Plant Materials and Crop Managements

Two high-yielding cassava genotypes, Rayong 72 (RY72; derived from the cross Rayong1 × Rayong5) and CMR35–91–63 (derived from the cross OMR29–20–118 × CMR25–105–112), which exhibit contrasting responses to drought, were selected for this study. RY72 is a widely cultivated commercial cassava cultivar in Thailand, characterized by high yield and drought tolerance, whereas CMR35–91–63 is a high-yielding genotype but is susceptible to drought stress [70,71]. Cassava was established on 9 May 2022 using stem cuttings approximately 20 cm in length obtained from 10-month-old plants. Stem cuttings were planted vertically on soil ridges, with approximately two-thirds of their length buried in the soil. Plants were spaced at 1.0 m × 1.0 m. The experiment was arranged as a split-plot within a randomized complete block design (RCBD) with four replications. Water management treatments were assigned to the main plots, while cassava genotypes were allocated to the subplots. Each subplot consisted of 56 plants arranged in 7 rows × 8 plants.

Land preparation and crop management practices followed the standard recommendations for cassava cultivation in experimental fields [72]. An overhead mini-sprinkler irrigation system was installed to provide supplementary water throughout the experimental period. Two water management regimes were imposed: (i) full irrigation (control), in which soil moisture was maintained near field capacity to a depth of 60 cm throughout the growing season, and (ii) drought treatment, in which irrigation was withheld for 60 days during the late growth stage (6–8 MAP) in the dry season (5 November 2022 to 5 January 2023), while plants were exposed to merely a few days of rainfall. Fertilizers were applied at 1 and 2MAP according to soil test recommendations and the nutrient requirements of cassava [73].

### 4.3. Data Collections

#### 4.3.1. Photosynthetic Performance

To compare the photosynthetic performance of cassava Rayong72 (RY72) and CMR35–91–63 grown under different water management regimes, photosynthetic measurements were conducted on three plants per replication at five developmental stages: 6, 7, 8, 9, and 12MAP. Chlorophyll fluorescence parameters, including the maximum quantum yield of photosystem II (Fv/Fm), effective quantum yield of PSII photochemistry (Φ PSII), and electron transport rate (ETR), were measured on the central lobe of the first fully expanded leaf (5th–6th leaf position from the apex). Measurements of Fv/Fm were performed at 05:00 h following dark adaptation under natural field conditions, whereas ΦPSII and ETR were recorded between 08:30 and 11:00 h under ambient sunlight using a Mini–PAM II Photosynthesis Yield Analyzer (Heinz Walz GmbH, Effeltrich, Germany).

Leaf gas exchange parameters, including net photosynthetic rate (Pn), stomatal conductance (gs), transpiration rate (Tr), and water use efficiency (WUE), were measured on the same leaves used for chlorophyll fluorescence assessments. Measurements were performed using a LI–6400XT portable photosynthesis system equipped with a red–blue LED light source and a standard 2 cm × 3 cm leaf chamber (LI–COR Biosciences, Lincoln, NE, USA). Environmental conditions within the leaf chamber were maintained at a light intensity of 1500 µmol photon m^−2^ s^−1^, a reference CO_2_ concentration of 400 µmol mol^−1^, and a leaf temperature of 30 ± 2 °C.

#### 4.3.2. Sugars and Starch Content of Leaf, Stem and Tuber

The first fully expanded leaves, stems, and tubers were sampled from cassava plants at 6, 7, 8, 9, and 12MAP. Samples were collected from one plant per replication (n = 4), cut into small pieces (approximately 0.1 g fresh weight, and stored at −20 °C until analysis of sugar and starch contents. Sugar extraction was performed following a modified method of Luo and Huang [74]. Frozen samples were finely ground, and 0.1 g of tissue was extracted with 80% (*v*/*v*) ethanol. The extracts were centrifuged at 12,000 rpm for 10 min at room temperature, and the supernatants were collected and stored at −20 °C until further analysis of total soluble sugars and reducing sugars.

Reducing sugar content (RS) was determined using the 3,5–dinitrosalicylic acid (DNS) method as described by Numan and Ford [75], with slight modifications. Briefly, 300 µL of extract was mixed with 300 µL of DNS reagent (containing 0.1 mM 3,5–dinitrosalicylic acid, 2.5 mM NaOH, and 30% potassium sodium tartrate) and 600 µL of distilled water. The reaction mixture was heated in a boiling water bath for 8 min and then cooled to room temperature. Absorbance was measured at 540 nm using a spectrophotometer (Hanon Advanced Technology Group Co., Ltd., Jinan, China). Reducing sugar concentration was expressed as mg g^−1^ DW based on a glucose standard curve.

Total soluble sugar content was quantified using the Anthrone method following Luo and Huang [74] with minor modifications. An aliquot of extract (10 µL) was diluted with 90 µL of distilled water and mixed with 600 µL of freshly prepared Anthrone reagent (0.5 mM Anthrone in 70% sulfuric acid). The reaction mixture was heated in a boiling water bath for 12 min and immediately cooled on ice to terminate the reaction. Absorbance was measured at 620 nm, and total sugar content was calculated from a glucose standard curve and expressed as mg g^−1^ DW. Non-reducing sugar content was estimated by subtracting reducing sugar content from total sugar content, following the method described by [76].

Starch content was determined following a modified method of Luo and Huang [74]. The residual pellet remaining after ethanol extraction of sugars was used for starch analysis. Briefly, the residue was washed with 500 µL of distilled water in a 1.5 mL microcentrifuge tube, followed by the addition of 650 µL of cold 52% perchloric acid. The mixture was thoroughly vortexed and incubated at room temperature for 30 min to facilitate starch extraction. Following incubation, samples were centrifuged at 13,000 rpm for 10 min, and the supernatants were collected. An aliquot of the extract was diluted with 100 µL of distilled water and mixed with 600 µL of freshly prepared Anthrone reagent. The reaction mixture was heated in a boiling water bath for 12 min and then rapidly cooled on ice to terminate the reaction. Absorbance was measured at 620 nm using a spectrophotometer (Hanon Advanced Technology Group Co., Ltd., Jinan, China). Soluble sugars (mg g^−1^ DW) were quantified using glucose as the standard, and starch content was calculated by comparison with a standard curve prepared using glucose standards.

#### 4.3.3. Malondialdehyde (MDA) and Hydrogen Peroxide (H_2_O_2_)

Malondialdehyde (MDA) content, an indicator of lipid peroxidation and oxidative membrane damage, was determined according to the method of Velikova et al. [77]. Fresh leaf tissue (0.1 g) was homogenized in 2.5 mL of 0.1% (*w*/*v*) trichloroacetic acid (TCA; Sigma-Aldrich, Milwaukee, WI, USA). The homogenate was centrifuged at 10,000× *g* for 20 min, and 0.5 mL of the resulting supernatant was mixed with 5.0 mL of 0.5% (*w*/*v*) thiobarbituric acid (TBA; Sigma-Aldrich, Milwaukee, WI, USA) prepared in 20% (*w*/*v*) TCA. The reaction mixture was heated in a boiling water bath for 10 min and then rapidly cooled in an ice bath for 5 min to terminate the reaction. After cooling, the samples were centrifuged at 10,000× *g* for 5 min. The absorbance of the supernatant was measured at 532 and 600 nm using a spectrophotometer (Hanon Advanced Technology Group Co., Ltd., Jinan, China). MDA content was determined using the extinction coefficient of the MDA-TBA product (155 mM^−1^ cm^−1^). The MDA concentration was calculated according to the formula: MDA (mM) = (A_532_ − A_600_)/155, where A_532_ and A_600_ represent the absorbance values measured at 532 and 600 nm, respectively.

Hydrogen peroxide (H_2_O_2_) content was determined following the method of Velikova et al. [77]. Fresh leaf tissue (0.1 g) was ground in liquid nitrogen and homogenized in 1 mL of 0.1% (*w*/*v*) trichloroacetic acid (TCA). The homogenate was centrifuged at 12,000 rpm for 15 min at 4 °C. After centrifugation, 0.5 mL of the supernatant was mixed with 0.5 mL of 10 mM potassium phosphate buffer (K_2_HPO_4_, pH 7.0) and 1.0 mL of 1 M potassium iodide (KI). The absorbance of the reaction mixture was measured at 390 nm using a spectrophotometer (Hanon Advanced Technology Group Co., Ltd., Jinan, China). Hydrogen peroxide concentration was determined from a standard curve prepared using known concentrations of H_2_O_2_ and expressed on a fresh weight basis.

#### 4.3.4. Antioxidant Enzyme Activity

For the determination of antioxidant enzyme activities, 0.5 g of fresh leaf tissue was homogenized in 5 mL of extraction buffer containing 10 mM potassium phosphate buffer (pH 7.0) and 4% (*w*/*v*) polyvinylpyrrolidone (PVP). The homogenate was centrifuged at 12,000× *g* for 15 min at 4 °C, and the resulting supernatant was immediately used for enzyme activity assays. All extraction procedures were conducted at 4 °C. Protein content in the crude enzyme extract was determined according to the method of Bradford [78]. Antioxidant enzyme activities were assayed following the procedures described by Pamuta et al. [79].

Catalase (CAT) activity was determined by mixing 100 μL of enzyme extract with a reaction mixture containing 1.8 mL of 50 mM potassium phosphate buffer (pH 7.0) and 100 μL of 0.5 M H_2_O_2_. The decrease in absorbance was monitored at 240 nm using a spectrophotometer at 1 min intervals for 3 min following the addition of H_2_O_2_. CAT activity was expressed as μmol H_2_O_2_ decomposed mg^−1^ protein min^−1^.

For peroxidase (POX) activity, the reaction mixture consisted of 3.0 mL of 0.1 M phosphate buffer (pH 7.0), 50 μL of 20 mM guaiacol, 100 μL of enzyme extract, and 30 μL of 12.3 mM H_2_O_2_. The reaction was initiated by the addition of H_2_O_2_, and the formation of tetraguaiacol (guaiacol dehydrogenation product, GDHP) was monitored by measuring the increase in absorbance at 436 nm using a spectrophotometer. POX activity was expressed as µmol GDHP decomposed mg^−1^ protein min^−1^.

Ascorbate peroxidase (APX) activity was assayed by mixing 100 μL of enzyme extract with a reaction mixture containing 1.7 mL of 50 mM potassium phosphate buffer (pH 7.0), 100 μL of 20 mM ascorbic acid, 50 μL of 0.5 M H_2_O_2_, and 50 μL of 16 mM EDTA. The decrease in absorbance was measured at 290 nm every 30 s for 2 min using a spectrophotometer. APX activity was expressed as μmol ascorbic acid oxidized mg^−1^ protein min^−1^.

#### 4.3.5. Growth and Yield

For growth assessments, four plants per genotype (one plant from each replication) were destructively sampled at 6, 7, 8, and 9 months after planting (MAP). At the final harvest (12MAP), 40 plants per genotype (10 plants from each replication) were sampled for yield determination. Following harvest, each plant was separated into leaves, petioles, stems, roots, and storage roots. Plant samples were subsequently weighed to determine growth in each plant component. Root sampling was conducted using the monolith method. Soil monoliths with a volume of approximately 18,000 cm^3^ (30 cm × 30 cm × 20 cm depth) were excavated, with the plant stem positioned at the center of the sampling area. For each plant, leaves, petioles, stems, roots and tuber were separated, dried at 80 °C for 48 h (BF 720, INDER GmbH (Headquarters), Tuttlingen, Germany), or until weight was constant and then weighed to obtain dry weight.

### 4.4. Data and Statistical Analysis

Data normality was assessed using the Shapiro-Wilk test [80] implemented in Statistix version 10 (Analytical Software, Tallahassee, FL, USA). Based on the distribution of the data, analysis of variance (ANOVA) was performed using a split-plot arrangement within a randomized complete block design (RCBD) to evaluate the effects of water treatments, cassava genotype, and their interaction on physiological traits, sugars and starch contents, antioxidant enzyme activity, growth, and yields. Water management was assigned to the main plots, whereas cassava genotypes were allocated to the subplots. Differences among cassava crop ages were analyzed using one-way ANOVA. Fisher’s Least Significant Difference (LSD) test was used for multiple comparisons of means at an alpha level of 0.05. All graphs were generated using SigmaPlot version 11.0 (Systat Software Inc., San Jose, CA, USA). To investigate the relationships among photosynthetic parameters [maximum quantum yield of PSII (Fv/Fm), effective quantum yield of PSII (ΦPSII), electron transport rate (ETR), net photosynthetic rate (Pn), stomatal conductance (gs), transpiration rate (Tr), and water use efficiency (WUE)], oxidative stress indicators (MDA and H_2_O_2_), antioxidant enzyme activities (CAT, POX, and APX), carbohydrate contents (RS, NRS and total sugar in leaves, stems, and tubers), and growth traits (fresh weight of leaves, petioles, stems, roots, and tubers) across the two cassava genotypes, Pearson’s correlation analysis and principal component analysis (PCA) were performed under both control and drought conditions. Statistical analyses were conducted using R version 4.5.2 [81] and RStudio version 2023.12.1 Build 402 [82].

## 5. Conclusions

The physiological, biochemical, and growth responses demonstrate that moderate drought during the tuber bulking stage had a greater impact on carbon partitioning and biomass accumulation than on photosynthetic capacity in cassava. Both genotypes maintained ΦPSII photochemistry, electron transport, stomatal function, and net photosynthesis rates throughout the drought period, indicating that the photosynthetic apparatus was highly resilient to water deficit at the late growth stage. Although drought-induced moderate oxidative stress, as evidenced by increased H_2_O_2_ and lipid peroxidation, both genotypes effectively activated antioxidant defense systems, particularly APX together with CAT and POX, and were thereby associated with limited oxidative damage and enabling rapid physiological recovery following rewatering. Drought primarily altered source-sink relationships, with assimilates being temporarily stored in stems while soluble sugars in storage roots declined, suggesting a shift in source-sink allocation rather than impaired carbon assimilation. Following rewatering, RY72 exhibited more effective remobilization of stored carbohydrates from stems to storage roots. These physiological and metabolic differences were ultimately reflected in plant productivity, as RY72 retained greater shoot and storage root biomass and experienced smaller reductions in total biomass and tuber yield under prolonged drought. Overall, the greater drought resilience of RY72 appears to result not from enhanced maintenance of photosynthesis alone, but from the coordinated regulation of photosynthetic function, antioxidant defense, source-sink carbohydrate partitioning and assimilate remobilization during recovery. This integrated physiological strategy enables RY72 to maintain carbon utilization and sustain higher productivity under prolonged drought during the tuber bulking stage compared with CMR35–91–63.

## Figures and Tables

**Table 1:** Monthly mean light intensity, air temperature, total rainfall, and number of rainy days recorded in a cassava field from May 2022 to May 2023. Cassava was planted on 9 May 2022 at the Field Crop Research Station, Faculty of Agriculture, Khon Kaen University, Thailand. Gray-shaded areas indicate the period during which irrigation was withheld for the drought treatment (0–60 days).

Month/Year	Crop Age	Seasons in Thailand	Light Intensity	Temperature (°C)			Total Rainfall	Rainy Day
	(Month)		(µmol Photon m^−2^ s^−1^)	max.	min.	mean	(mm)	(day)
May, 2022	0	late hot	465	30.9	23.1	27.0	179.5	30
June, 2022	1	rainy	555	30.7	23.9	27.3	141.9	28
July, 2022	2	rainy	480	29.9	24.3	27.1	359.9	31
August, 2022	3	rainy	462	29.4	23.7	26.6	292.6	31
September, 2022	4	rainy	425	28.9	23.7	26.3	417.5	30
October, 2022	5	late rainy	472	27.5	20.6	24.0	127.2	15
November, 2022	6	cool	466	28.8	20.7	24.7	51.4	13
December, 2022	7	cool	472	27.3	15.3	21.3	2.7	1
January, 2023	8	cool	473	29.1	15.0	22.1	7.2	3
February, 2023	9	late cool	487	33.1	19.5	26.3	15.9	9
March, 2023	10	hot	548	36.3	22.7	29.5	17.8	7
April, 2023	11	hot	517	38.7	26.7	32.7	37.1	15
May, 2023	12	late hot	548	36.4	26.2	31.3	128.3	22

**Table 2:** Physicochemical properties of soil at pre-planting in the experimental field at depths of 0–30 cm and 30–60 cm, and soil moisture (%) in the control and drought fields at the different crop ages.

	**Soil Physicochemical Properties**			
	**0–30 cm Soil Depth**		**30–60 cm Soil Depth**	
Sand (%)	73.73		73.44	
Silt (%)	21.08		19.26	
Clay (%)	5.19		7.3	
Soil Texture	Sandy Loam		Sandy Loam	
Total N (%)	0.032		0.034	
Available P (mg kg^−1^)	28.13		18.75	
Exchangeable K (mg kg^−1^)	27		23.94	
Organic matter (%)	0.55		0.58	
CEC (cmol kg^−1^)	2.5		3.2	
pH (1:1 H_2_O)	5.42		5.51	
EC (dS m^−1^)	0.038		0.029	
**Crop Age**	**Soil Moisture (%)**			
	**0–30 cm Soil Depth**		**30–60 cm Soil Depth**	
	**Control**	**Drought**	**Control**	**Drought**
6MAP	12.39	12.12	12.50	12.50
7MAP	11.67 **	5.95	9.70 *	8.65
8MAP	13.15 **	3.98	8.02 **	5.86
9MAP	11.42	11.26	7.03	6.49

N: nitrogen; P: phosphorus; K: potassium; EC: electrical conductivity; CEC: cation exchange capacity; OM: organic matter; MAP: month after planting; DAS: day after stress; DAR: day after recovery. Significant differences in soil moisture between the control and drought conditions are indicated by * (*p* < 0.05) and ** (*p* < 0.01).

## Data Availability

All data supporting the findings of this study are available within the paper.

## Supplementary Materials

The following supporting information can be downloaded at https://www.mdpi.com/article/10.3390/plants15193003/s1, Table S1: Chlorophyll fluorescence parameters; Table S2: Photosynthetic parameters; Table S3: Leaf sugar (reducing, non-reducing and total sugar) and starch contents; Table S4: Stem sugar (reducing, non-reducing and total sugar) and starch contents; Table S5: Tuber sugar (reducing, non-reducing and total sugar) and starch contents; Table S6: Malondialdehyde (MDA), hydrogen peroxide (H_2_O_2_), and antioxidant activity including catalase (CAT), peroxidase (POX), and ascorbate peroxidase (APX); Table S7: Leaf, petiole, stem, root, tuber and total fresh weigh (DW) of cassava plants; Table S8: Spit-plot ANOVA.

## Abbreviations

The following abbreviations are used in this manuscript:

APX	ascorbate peroxidase
ANOVA	analysis of variance
CAT	catalase
CMR	Cross Manihot Rayong
CO_2_	carbon dioxide
DAR	Days after recovery
DAS	days after stress
DNS	3,5-dinitrosalicylic acid
DW	Dry weight
ETR	electron transport rate
Fv/Fm	maximum quantum yield of PSII
FW	Fresh weight
GDHP	guaiacol dehydrogenation product
gs	stomatal conductance
H_2_O_2_	hydrogen peroxide
LSD	Least Significant Difference
MAP	month after planting
MDA	Malondialdehyde
NPQ	non-photochemical quenching
NRS	non-reducing sugar
PC	principal component
PCA	principal component analysis
Pn	Net photosynthesis
POX	peroxidase
PVP	polyvinylpyrrolidone
RCBD	randomized complete block design
ROS	reactive oxygen species
RS	reducing Sugar
RY	Rayong
SE	Standard error
TBA	thiobarbituric acid
TCA	trichloroacetic acid
Tr	transpiration rate
WUE	water use efficiency
ΦPSII	effective quantum yield of PSII
