# Supplementary material for: Growth, Antioxidant, and Physiological Responses Associated with Tuber Yield in Cassava Under Irrigation and Late-Season Drought Conditions in a Tropical Savanna Climate
Source: Plants (Basel). 2026 Sep 30;15(19):3003. doi: 10.3390/plants15193003 (PMC13667232; doi:10.3390/plants15193003)
Supplement: Supplementary file 1 [file plants-15-03003-s001.zip › plants-4553595-supplementary.pdf]

Supplementary Tables for

Santanoo et al. (2026)

Growth, Antioxidant, and Physiological Responses Associated with Tuber Yield in Cassava under Irrigation and Late–Season Drought Conditions in a Tropical Savanna Climate

Table S1. Chlorophyll fluorescence parameters, including the maximum quantum yield of PSII (Fv/Fm, (**A**)), effective quantum yield of PSII photochemistry (ΦPSII), electron transport rate (ETR), and non–photochemical quenching (NPQ) of RY72 and CMR35–91–63 measured at 6, 7, 8, 9, and 12 months after planting (MAP). Plants were grown under well–watered conditions (continuous irrigation from planting to 12MAP) and drought conditions (irrigation withheld for 60 days during the dry season from 6 to 8MAP, followed by rewatering until 12MAP). Different uppercase letters indicate significant differences among crop ages ($p < 0.05$ or $p < 0.01$), whereas different lowercase letters indicate significant differences between genotypes within each crop age. Asterisks (*) denote values that are significantly different ($p < 0.05$) from the control treatment.

Parameter	Crop age	RY72		CMR35–91–63		Increase /decrease from control (%)	
		Control	Drought	Control	Drought	RY72	CMR35–91–63
Fv/Fm	6MAP	0.814 aB	0.826 aAB	0.821 aA	0.821 aB	1	0
	7MAP	0.822 aB	0.821 aB	0.817 aA	0.821 aB	0	0
	8MAP	0.789 aC	0.778 aC	0.773 aB	0.784 aC	−1	1
	9MAP	0.815 aB	0.814 aB	0.803 aA	0.810 aB	0	4
	12MAP	0.840 aA	0.841 aA	0.828 aA	0.837 aA	0	−2
	F–test	14.28	19.22	5.47	14.41		
	P–value	< 0.01	< 0.01	< 0.01	< 0.01		
ΦPSII	6MAP	0.243 aB	0.213 aB	0.254 aC	0.246 aB	−12	−3
	7MAP	0.308 aB	0.274 aB	0.314 aB	0.314 aB	−11	0
	8MAP	0.243 aB	0.236 aB	0.295 aBC	0.255 aB	−1	−14
	9MAP	0.296 aB	0.244 aB	0.275 aBC	0.304 aB	−18	10
	12MAP	0.453 aA	0.429 abA	0.440 aA	0.390 bA	−5	−11*
	F–test	11.8	6.84	15.98	6.42		
	P–value	< 0.01	< 0.01	< 0.01	< 0.01		
ETR (μmol e– m^{-2} s^{-1})	6MAP	143 aB	119 a	140 a	134 a	−17	−4
	7MAP	159 aAB	148 a	170 a	161 a	−7	−5
	8MAP	140 aB	131 a	144 a	139 a	−2	−4
	9MAP	156 aAB	140 a	159 a	170 a	−10	7
	12MAP	186 aA	176 a	181 a	171 a	−5	−6
	F–test	3.86	1.4	2.27	2		
	P–value	< 0.05	NS	NS	NS		
NPQ	6MAP	1.431 aB	1.388 a	1.862 aA	1.288 a	−3	−31
	7MAP	1.038 aC	1.400 a	1.196 aB	1.243 a	35	4
	8MAP	1.937 aA	1.716 a	1.288 aB	1.709 a	−11	33
	9MAP	1.601 aAB	1.114 a	1.373 aAB	1.227 a	−30	−11
	12MAP	0.969 aC	0.987 a	0.842 aB	1.020 a	2	21
	F–test	11.7	1.4	4.25	1.16		
	P–value	< 0.01	NS	< 0.05	NS		

F– and *P*–values for differences in the values of each trait among crop ages under each water treatment.
NS indicates a non–significant difference.

Table S2. Photosynthetic parameters including net photosynthesis (Pn), stomatal conductance (gs), transpiration rate (Tr), and water use efficiency (WUE) of RY72 and CMR35–91–63 measured at 6, 7, 8, 9, and 12 months after planting (MAP). Plants were grown under well–watered conditions (continuous irrigation from planting to 12MAP) and drought conditions (irrigation withheld for 60 days during the dry season from 6 to 8MAP, followed by rewatering until 12MAP). Different uppercase letters indicate significant differences among crop ages ($p < 0.05$ or $p < 0.01$), whereas different lowercase letters indicate significant differences between genotypes within each crop age. Asterisks (*) denote values that are significantly different ($p < 0.05$) from the control treatment.

**Parameter**	**Crop age**	**RY72**		**CMR35–91–63**		**Increase or decrease from control (%)**	
		**Control**	**Drought**	**Control**	**Drought**	**RY72**	**CMR35–91–63**
Pn (μmol CO_2 m^{-2} s^{-1})	6MAP	24.56 abA	24.48 bA	25.60 abA	26.38 aA	0	3
	7MAP	19.98 aAB	16.99 aBC	19.75 aB	15.05 aBC	–15	–24
	8MAP	14.48 aB	11.96 aC	17.37 aB	11.96 aC	–17	–31
	9MAP	20.37 aAB	21.50 aAB	18.26 aB	18.27 aB	6	0
	12MAP	15.33 aB	15.83 aC	12.31 abC	9.86 bC	3	–20
	F–test	8.71	15.15	35.17	25.64		
	P–value	< 0.01	< 0.01	< 0.01	< 0.01		
gs (mol H_2O m^{-2} s^{-1})	6MAP	0.38 bA	0.36 bA	0.39 bA	0.44 aA	–5	11*
	7MAP	0.07 aB	0.06 aC	0.07 aB	0.07 aB	–11	–7
	8MAP	0.09 aB	0.08 aC	0.12 aB	0.08 aB	–6	–33
	9MAP	0.14 aB	0.15 aBC	0.17 aB	0.10 aB	4	–17
	12MAP	0.32 aA	0.24 aAB	0.23 aAB	0.18 aB	–25	–21
	F–test	15.20	12.32	9.69	25.36		
	P–value	< 0.01	< 0.01	< 0.01	< 0.01		
Tr (mmol H_2O m^{-2} s^{-1})	6MAP	6.31 aA	5.80 aA	6.76 aA	6.39 aA	–8	–5
	7MAP	2.11 aB	1.79 aC	2.12 aC	1.83 aB	–15	–14
	8MAP	2.78 aB	2.63 aC	3.63 aBC	2.54 aB	–5	–30
	9MAP	3.38 aB	3.32 aBC	3.461 aBC	2.71 aB	–2	–22
	12MAP	5.27 aA	4.62 aAB	4.71 aB	3.92 aB	–12	–17
	F–test	18.86	12.24	14.85	11.94		
	P–value	< 0.01	< 0.01	< 0.01	< 0.01		
WUE (μmol CO_2 mmol H_2O^{-1})	6MAP	3.89 aBC	4.22 aB	3.79 aB	4.13 aBC	9	9
	7MAP	9.66 aA	10.48 aA	9.86 aA	9.99 aA	9	1
	8MAP	5.22 aBC	4.60 aB	4.86 aB	4.66 aB	–12	–4
	9MAP	6.67 aAB	6.91 aAB	5.85 aB	7.02 aAB	4	20
	12MAP	2.89 aC	3.74 aB	2.71 aB	2.79 aC	29	3
	F–test	13.53	11.07	11.73	9.2		
	P–value	< 0.01	< 0.01	< 0.01	< 0.01		

F– and *P*–values for differences in the values of each trait among crop ages under each water treatment.

Table S3. Leaf sugar (reducing, non–reducing and total sugar) and starch contration of RY72 and CMR35–91–63 measured at 6, 7, 8, 9, and 12 months after planting (MAP). Plants were grown under well–watered conditions (continuous irrigation from planting to 12MAP) and drought conditions (irrigation withheld for 60 days during the dry season from 6 to 8MAP, followed by rewatering until 12MAP). Different uppercase letters indicate significant differences among crop ages ($p < 0.05$ or $p < 0.01$), whereas different lowercase letters indicate significant differences between genotypes within each crop age. Asterisks (*) denote values that are significantly different ($p < 0.05$) from the control treatment.

Leaf	Crop age	RY72		CMR35–91–63		Increase or decrease from control (%)	
		Control	Drought	Control	Drought	RY72	CMR35–91–63
Reducing sugar (mg g⁻¹ DW)	6MAP	24.69 aB	43.81 aA	39.27 aA	35.35 aA	77	−10
	7MAP	41.89 aA	25.36 bB	30.84 abA	24.06 bA	−39*	−22
	8MAP	24.72 aB	27.34 aB	33.32 aA	30.60 aA	11	−8
	9MAP	23.93 aB	22.33 aB	24.79 aAB	20.48 aA	−7	−17
	12MAP	18.14 aB	34.66 aAB	15.37 aB	23.42 aA	91	52
	F–test	4.20	3.14	3.33	0.80		
	P–value	< 0.05	< 0.05	< 0.05	NS		
Non–reducing sugar (mg g⁻¹ DW)	6MAP	81.45 aA	55.92 bA	59.96 bA	43.20 bA	−31*	−28
	7MAP	47.62 bB	57.33 abA	74.36 aA	54.88 abA	20	−26
	8MAP	48.31 aB	33.18 aA	52.85 aA	39.56 aA	−31	−25
	9MAP	58.70 aB	50.68 aA	56.21 aA	55.87 aA	−14	−1
	12MAP	57.65 aB	39.73 aA	38.59 aA	47.38 aA	−31	23
	F–test	4.33	1.68	2.29	0.93		
	P–value	< 0.05	NS	NS	NS		
Total sugar (mg g⁻¹ DW)	6MAP	106.14 aA	99.73 abA	99.23 abA	78.55 bA	−6	−21
	7MAP	89.51 abAB	82.69 bAB	105.20 aA	78.94 bA	−8	−25*
	8MAP	73.04 aB	60.52 aC	86.17 aA	70.16 aA	−17	−19
	9MAP	82.63 aB	73.01 aBC	81.01 aA	76.35 aA	−12	−6
	12MAP	75.78 aB	74.39 aBC	53.96 bB	70.80 abA	−2	31
	F–test	4.18	3.90	5.79	0.27		
	P–value	< 0.05	< 0.05	< 0.01	NS		
Starch concentration (mg g⁻¹ DW)	6MAP	30.28 aB	37.37 aB	31.42 aB	31.78 aB	23	1
	7MAP	29.81 aB	37.28 aB	33.55 aB	32.20 aB	25	−4
	8MAP	31.41 aB	36.68 aB	51.10 aAB	40.92 aB	17	−20
	9MAP	62.13 aA	75.40 aA	71.23 aA	68.70 aA	21	−4
	12MAP	27.01 aB	25.92 aB	23.55 aB	27.29 aB	−4	16
	F–test	19.57	8.75	3.94	9.42		
	P–value	< 0.01	< 0.01	< 0.05	< 0.01		

F– and *P*–values for differences in the values of each trait among crop ages under each water treatment.
NS indicates a non–significant difference.

Table S4. Stem sugar (reducing, non–reducing and total sugar) and starch contration of RY72 and CMR35–91–63 measured at 6, 7, 8, 9, and 12 months after planting (MAP). Plants were grown under well–watered conditions (continuous irrigation from planting to 12MAP) and drought conditions (irrigation withheld for 60 days during the dry season from 6 to 8MAP, followed by rewatering until 12MAP). Different uppercase letters indicate significant differences among crop ages ($p < 0.05$ or $p < 0.01$), whereas different lowercase letters indicate significant differences between genotypes within each crop age. Asterisks (*) denote values that are significantly different ($p < 0.05$) from the control treatment.

Stem	Crop age	RY72		CMR35–91–63		Increase or decrease from control (%)	
		Control	Drought	Control	Drought	RY72	CMR35–91–63
Reducing sugar (mg g^{-1} DW)	6MAP	12.97 aA	18.48 aA	15.79 aA	12.02 aA	42	−24
	7MAP	4.55 aBC	3.72 aB	7.00 aA	3.18 aB	−18	−55
	8MAP	6.00 aBC	3.18 aB	6.28 aA	5.33 aB	−47	−15
	9MAP	8.52 aB	6.28 aB	5.63 aA	5.02 aB	−26	−11
	12MAP	4.29 aC	17.33 aA	5.87 aA	16.05 aA	304	173
	F–test	6.84	7.90	1.37	6.62		
	P–value	< 0.01	< 0.01	0.2903	< 0.01		
Non–reducing sugar (mg g^{-1} DW)	6MAP	24.12 aB	41.14 aB	24.41 aB	38.50 aA	71	58
	7MAP	26.47 aB	24.42 aB	27.03 aB	28.71 aAB	−8	6
	8MAP	20.32 aB	18.24 abB	7.64 bC	13.87 abB	−10	82
	9MAP	12.75 cB	32.65 aB	16.42 cBC	24.60 bAB	156*	50*
	12MAP	45.83 abA	66.57 aA	39.59 abA	34.06 bA	45	−14
	F–test	6.80	5.00	8.9	3.22		
	P–value	< 0.01	< 0.01	< 0.01	< 0.01		
Total sugar (mg g^{-1} DW)	6MAP	37.09 aAB	59.61 aB	40.19 aAB	50.53 aA	61	26
	7MAP	31.03 aBC	28.14 aC	34.02 aA–C	31.90 aBC	−9	−6
	8MAP	26.32 aBC	21.42 abC	13.92 bC	19.20 abC	−19	38
	9MAP	21.27 bC	38.93 aBC	22.06 bBC	29.62 bC	83*	34
	12MAP	50.12 bA	83.91 aA	45.46 bA	50.11 bAB	67*	10
	F–test	5.75	10.80	3.62	5.12		
	P–value	< 0.01	< 0.01	< 0.05	< 0.01		
Starch concentration (mg g^{-1} DW)	6MAP	39.81 bB	58.19 abB	98.23 abA	148.71 aA	46	51
	7MAP	101.80 bAB	97.63 bB	141.46 abA	177.35 aA	−4	25
	8MAP	119.01 aA	90.37 aB	88.15 aA	108.00 aA	−24	23
	9MAP	128.33 bA	217.63 aA	185.26 aA	234.47 aA	70	27
	12MAP	135.04 aA	183.32 aA	142.53 aA	129.38 aA	36	−9
	F–test	2.92	8.22	2.91	1.78		
	P–value	< 0.05	< 0.01	NS	NS		

F– and *P*–values for differences in the values of each trait among crop ages under each water treatment.
NS indicates a non–significant difference.

Table S5. Tuber sugar (reducing, non−reducing and total sugar) and starch contration of Rayong 72 (RY72) and CMR35−91−63 at the crop age 6, 7, 8, 9, and 12−months after planting (MAP). Sugars and starch of tuber were measured in cassava growing under the control (continuous irrigation from 0MAP to 12MAP and drought (irrigation was withheld for 60 days in the dry season during 6MAP to 8MAP then rewatered until 12MAP) treatment. Different capital letters indicated significant ($p < 0.05$ and $p < 0.01$) differences among crop age, whereas those among genotypes are denoted with different lowercase letters in each crop age. Significant increased or decreased values relative to the control are noted by *.

Tuber	Crop age	RY72		CMR35−91−63		Increase or decrease from control (%)	
		Control	Drought	Control	Drought	RY72	CMR35−91−63
Reducing sugar (mg g^{-1} DW)	6MAP	19.88 aAB	16.21 aA	22.85 aA	20.51 aA	−18	−10
	7MAP	21.34 aAB	1.34 bD	18.13 aA	2.49 bB	−94*	−86*
	8MAP	10.95 aBC	6.19 aCD	7.12 aA	7.55 aB	−43	6
	9MAP	6.05 aC	9.02 aBC	15.10 aA	3.44 aB	49	−77
	12MAP	21.61 aA	14.14 aAB	14.21 aA	22.50 aA	−35	58
	F−test	4.06	9.43	1.01	5.82		
	P−value	< 0.05	< 0.01	NS	< 0.01		
Non−reducing sugar (mg g^{-1} DW)	6MAP	45.15 aBC	34.49 bB	28.75 bC	27.28 bA	−24	−5
	7MAP	49.00 aB	27.28 aB	39.52 aB	25.44 aA	−44	−36
	8MAP	29.97 aC	18.52 aB	22.31 aC	22.70 aA	−38	2
	9MAP	33.15 aBC	34.74 aB	26.68 aC	30.72 aA	5	15
	12MAP	71.55 abA	93.07 aA	55.78 bcA	41.83 cA	30	−25
	F−test	7.05	23.87	15.23	1.50		
	P−value	< 0.01	< 0.01	< 0.01	NS		
Total sugar (mg g^{-1} DW)	6MAP	65.03 aBC	50.71 aB	51.59 aAB	47.79 aB	−22	−7
	7MAP	70.34 aAB	28.62 bCD	57.64 aAB	27.93 bC	−59*	−52*
	8MAP	40.91 aC	24.71 aD	29.42 aC	30.25 aC	−40	3
	9MAP	39.20 aC	43.76 aBC	41.78 aBC	34.16 aBC	12	−18
	12MAP	93.16 aA	107.20 aA	69.99 bA	64.33 bA	15	−8
	F−test	6.41	36.82	6.07	8.48		
	P−value	< 0.01	< 0.01	< 0.01	< 0.01		
Starch concentration (mg g^{-1} DW)	6MAP	440.80 abA	484.13 aC	318.86 bB	513.17 aC	10	61*
	7MAP	407.11 aA	443.55 aC	430.11 aB	468.56 aC	9	9
	8MAP	617.42 aA	993.76 aA	697.76 aA	897.37 aA	61	29
	9MAP	602.33 aA	755.75 aB	652.47 aA	813.38 aB	25	25
	12MAP	422.00 aA	445.08 aC	436.41 aB	487.23 aC	5	12
	F−test	1.78	19.67	16.06	13.18		
	P−value	NS	< 0.01	< 0.01	< 0.01		

F− and *P*−values for differences in the values of each trait among crop ages under each water treatment.
NS indicates a non−significant difference.

Table S6. Malondialdehyde (MDA), hydrogen peroxide (H_2O_2), and antioxidant activity including catalase (CAT), peroxidase (POX), and ascorbate peroxidase (APX) of RY72 and CMR35–91–63 measured at 6, 7, 8 and 9 months after planting (MAP). Plants were grown under well–watered conditions (continuous irrigation from planting to 12MAP) and drought conditions (irrigation withheld for 60 days during the dry season from 6 to 8MAP, followed by rewatering until 12MAP). Different uppercase letters indicate significant differences among crop ages ($p < 0.05$ or $p < 0.01$), whereas different lowercase letters indicate significant differences between genotypes within each crop age. Asterisks (*) denote values that are significantly different ($p < 0.05$) from the control treatment.

Leaf	Crop age	RY72		CMR35–91–63		Increase or decrease from control (%)	
		Control	Drought	Control	Drought	RY72	CMR35–91–63
MDA (nmol g^{-1} FW)	6MAP	0.136	0.140 C	0.108	0.118 B	3	9
	7MAP	0.143 b	0.211 aB	0.123 b	0.144 bB	48*	17
	8MAP	0.138 c	0.244 aA	0.122 c	0.206 bA	77*	69*
	9MAP	0.132 ab	0.140 aC	0.105 b	0.106 bB	6	1
	F–test	0.53	35.66	1.83	7.70		
	P–value	NS	< 0.01	NS	< 0.01		
H_2O_2 (µmol g^{-1} FW)	6MAP	0.655 a	0.696 aB	0.346 bA	0.270 bB	6	−22
	7MAP	0.596 b	1.190 aA	0.308 cB	0.374 cB	100*	22
	8MAP	0.597 b	1.358 aA	0.296 cB	0.729 bA	127*	146*
	9MAP	0.740 a	0.688 aB	0.258 bC	0.298 bB	−7	15
	F–test	1.83	11.41	18.25	13.12		
	P–value	NS	< 0.01	< 0.01	< 0.01		
CAT (µmol H_2O_2 decomposed mg^{-1} protein min^{-1})	6MAP	14.136 c	15.654 cB	43.474 bAB	57.435 aD	11	32
	7MAP	16.595 c	24.353 cB	35.945 bB	81.345 aB	47	126*
	8MAP	14.654 d	40.510 cA	56.712 bA	91.326 aA	176*	61*
	9MAP	13.638 b	17.220 bB	56.675 aA	67.988 aC	26	20
	F–test	0.29	11.25	4.60	70.61		
	P–value	NS	< 0.01	< 0.05	< 0.01		
POX (µmol GDHP decomposed mg^{-1} protein min^{-1})	6MAP	0.118 A	0.132 BC	0.103 B	0.122 B	12	18
	7MAP	0.151 A	0.243 B	0.202 A	0.260 B	60	29
	8MAP	0.185 bA	0.436 bA	0.120 bB	0.762 aA	136	537*
	9MAP	0.011 bB	0.044 bC	0.032 bC	0.107 aB	283	231
	F–test	7.81	10.03	9.66	17.01		
	P–value	< 0.01	< 0.01	< 0.01	< 0.01		
APX (µmol ascorbic acid decomposed mg^{-1} protein min^{-1})	6MAP	23.993 a	25.641 aC	5.314 b	8.254 bC	7	55
	7MAP	16.315 bc	40.856 aB	9.821 c	25.701 abB	150*	162*
	8MAP	18.980 c	64.448 aA	13.405 c	51.742 bA	240*	286*
	9MAP	24.655 a	26.647 aC	12.434 b	11.994 bC	8	−4
	F–test	2.17	15.77	2.72	23.77		
	P–value	NS	< 0.01	NS	< 0.01		

F– and *P*–values for differences in the values of each trait among crop ages under each water treatment.
NS indicates a non–significant difference.

Table S7. Leaf, petiole, stem, root, tuber dry weight (DW) and starch content, and total DW of RY72 and CMR35–91–63 measured at 6, 7, 8, 9, and 12 months after planting (MAP). Plants were grown under well–watered conditions (continuous irrigation from planting to 12MAP) and drought conditions (irrigation withheld for 60 days during the dry season from 6 to 8MAP, followed by rewatering until 12MAP). Different uppercase letters indicate significant differences among crop ages ($p < 0.05$ or $p < 0.01$), whereas different lowercase letters indicate significant differences between genotypes within each crop age. Asterisks (*) denote values that are significantly different ($p < 0.05$) from the control treatment.

Parameter	Crop age	RY72		CMR35–91–63		Increase/decrease from control (%)	
		Control	Drought	Control	Drought	RY72	CMR35–91–63
Leaf DW (g plant^{-1})	6MAP	105 bA	128 abAB	179 aA	152 abB	22	–15
	7MAP	137 bAB	145 bA	108 bA	357 aA	6	232*
	8MAP	58 aD	95 aA–C	126 aA	146 aB	64	16
	9MAP	68 bCD	48 bC	168 aA	71 bC	–29	–58*
	12MAP	150 aA	76 bBC	174 aA	81 bC	–50*	–54*
	F–test	7.50	4.36	0.90	38.68		
	P–value	< 0.01	< 0.05	NS	< 0.01		
Petiole DW (g plant^{-1})	6MAP	33 aAB	46 aA	43 aA	49 aB	38	14
	7MAP	44 bA	45 bAB	43 bA	95 aA	1	120*
	8MAP	12 cC	29 bcBC	63 aA	41 bBC	144	–35*
	9MAP	20 abBC	14 bC	52 aA	20 abC	–31	–61
	12MAP	44 abA	27 cC	49 aA	30 bcBC	–39*	–39*
	F–test	9.94	6.23	1.22	11.50		
	P–value	< 0.01	< 0.01	NS	< 0.01		
Stem DW (g plant^{-1})	6MAP	302 aA	293 aB	533 aB	438 aB	–3	–18
	7MAP	455 bA	467 bAB	610 bB	1457 aA	3	139*
	8MAP	311 cA	605 bcA	934 abAB	1205 aA	95	29
	9MAP	540 bA	610 bA	1357 aA	686 bB	13	–49*
	12MAP	670 abA	273 bB	1124 aA	430 bB	–59	–62*
	F–test	1.79	5.06	4.44	22.49		
	P–value	NS	< 0.01	< 0.05	< 0.01		
Root DW (g plant^{-1})	6MAP	2 cA	14 aA	7 bcA	10 abAB	581	45
	7MAP	5 abA	1 bB	10 abA	11 aA	–84	3
	8MAP	3 bA	3 bB	13 aA	4 bBC	–9	–70*
	9MAP	3 bcA	2 cB	9 aA	7 abA–C	–44	–19
	12MAP	3 bA	1 bB	10 aA	1 bC	–82	–85*
	F–test	1.59	35.37	2.12	3.25		
	P–value	NS	< 0.01	NS	< 0.05		
Tuber DW (g plant^{-1})	6MAP	641 aB	706 aC	757 aB	692 aB	10	–9
	7MAP	1673 aA	1546 aAB	806 bB	1559 aA	–8	94*
	8MAP	2232 aA	1975 aA	2630 aA	949 bB	–12	–64*
	9MAP	1824 aA	1966 aA	1758 abAB	1107 bB	8	–37

Parameter	Crop age	RY72		CMR35−91−63		Increase/decrease from control (%)	
		Control	Drought	Control	Drought	RY72	CMR35−91−63
	12MAP	2506 aA	1086 bBC	2537 aA	851 bB	–57*	–66*
	F−test	6.10	5.24	5.74	5.22		
	P−value	< 0.01	< 0.01	< 0.01	< 0.01		
Tuber starch content (g plant⁻¹)	6MAP	270 aC	340 aB	246 aC	342 aB	26	39
	7MAP	694 aBC	681 aB	337 bC	729 aAB	−2	117*
	8MAP	1375 aA	1913 aA	1726 aA	851 bA	39	−51*
	9MAP	1139 aB	1489 aA	1143 aB	937 aA	31	−18
	12MAP	1060 aB	474 bB	1083 aB	421 bB	−55*	−61*
	F−test	7.68	13.19	11.42	3.20		
	P−value	< 0.01	< 0.01	< 0.01	< 0.05		
Total DW (g plant⁻¹)	6MAP	1092 aB	1082 aC	1503 aB	1340 aC	–1	–11
	7MAP	2309 bA	2225 bAB	1720 bB	3491 aA	–4	103*
	8MAP	2616 aA	2721 aA	3754 aA	2333 aB	4	–38
	9MAP	2453 abA	2681 abA	3344 aA	1897 bB	9	–43
	12MAP	3373 aA	1462 bBC	3894 aA	1402 bC	–57*	–64*
	F−test	4.40	8.05	4.78	29.80		
	P−value	< 0.05	< 0.01	< 0.05	< 0.01		

F– and *P*–values for differences in the values of each trait among crop ages under each water treatment.
NS indicates a non–significant difference.

Table S8. Spit–plot ANOVA showing levels of significant differences ($p < 0.05$ and $p < 0.01$) for chlorophyll fluorescence (Fv/Fm, ΦPSII, ETR and NPQ), leaf gas exchange (Pn, gs, Tr, and WUE), **s**ugars (reducing, non−reducing and total sugar) and starch of leaf, stem and tuber, malondialdehyde (MDA), hydrogen peroxide (H_2O_2), and antioxidant activity including catalase (CAT), peroxidase (POX), and ascorbate peroxidase (APX), and dry weight (DW) of leaves, petioles, stem, root, tuber, tatotal DW **a**nd tuber starch content of cassava plants Rayong 72 (RY72) and CMR35−91−63 at the crop age 6, 7, 8, 9, and 12−months after planting (MAP).

Parameter	Water condition (W)				Cassava genotype (G)				W x G			
	df	MS	*F*–test	*P*–value	*df*	MS	*F*–test	*P*–value	*df*	MS	*F*–test	*P*–value
Fv/Fm–6MAP	1	0.000	4.26	0.131	1	0.000	0.03	0.858	1	0.000	1.03	0.349
Fv/Fm–7MAP	1	0.000	0.04	0.855	1	0.000	0.30	0.603	1	0.000	0.23	0.645
Fv/Fm–8MAP	1	0.000	0.00	1.000	1	0.000	0.49	0.509	1	0.001	2.17	0.191
Fv/Fm–9MAP	1	0.000	0.12	0.750	1	0.000	3.55	0.108	1	0.000	0.59	0.470
Fv/Fm–12MAP	1	0.000	0.72	0.458	1	0.000	1.03	0.349	1	0.000	0.34	0.581
ΦPSII–6MAP	1	0.001	1.07	0.377	1	0.001	1.18	0.319	1	0.001	1.18	0.319
ΦPSII–7MAP	1	0.001	0.85	0.425	1	0.002	2.39	0.173	1	0.001	1.24	0.308
ΦPSII–8MAP	1	0.002	0.30	0.619	1	0.005	1.65	0.246	1	0.001	0.34	0.582
ΦPSII–9MAP	1	0.001	0.34	0.599	1	0.001	0.40	0.548	1	0.006	1.72	0.238
ΦPSII–12MAP	1	0.005	24.0	0.016	1	0.002	3.36	0.116	1	0.001	0.91	0.377
ETR–6MAP	1	841.0	1.80	0.272	1	156.2	0.27	0.619	1	342.2	0.60	0.467
ETR–7MAP	1	430.5	1.22	0.350	1	564.0	2.21	0.187	1	7.560	0.03	0.868
ETR–8MAP	1	196.0	0.10	0.769	1	156.2	0.21	0.661	1	9.000	0.01	0.915
ETR–9MAP	1	33.06	0.08	0.793	1	1040	0.70	0.434	1	742.5	0.50	0.505
ETR–12MAP	1	400.0	0.81	0.434	1	81.00	1.05	0.345	1	0.250	0.00	0.956
NPQ–6MAP	1	0.381	0.88	0.416	1	0.109	2.15	0.192	1	0.281	5.52	0.057
NPQ–7MAP	1	0.166	2.82	0.191	1	0.000	0.00	0.998	1	.0992	0.66	0.446
NPQ–8MAP	1	0.009	0.05	0.845	1	0.575	5.40	0.059	1	0.555	5.21	0.062
NPQ–9MAP	1	0.400	0.83	0.429	1	0.013	5.14	0.064	1	0.116	45.28	0.001
NPQ–12MAP	1	0.038	2.91	0.186	1	0.008	0.22	0.654	1	0.025	0.63	0.456
Pn–6MAP	1	0.490	0.42	0.563	1	8.642	9.96	0.019	1	0.767	0.88	0.383
Pn –7MAP	1	59.09	3.97	0.140	1	4.728	1.40	0.282	1	2.953	0.87	0.386
Pn –8MAP	1	62.87	3.60	0.154	1	8.389	4.15	0.087	1	8.366	4.14	0.088

Parameter	Water condition (W)				Cassava genotype (G)				W x G			
	df	MS	*F*–test	*P*–value	*df*	MS	*F*–test	*P*–value	*df*	MS	*F*–test	*P*–value
Pn –9MAP	1	1.298	0.16	0.713	1	28.50	4.42	0.080	1	1.272	0.20	0.672
Pn –12MAP	1	3.801	4.94	0.112	1	80.83	8.85	0.024	1	8.727	0.96	0.366
gs–6MAP	1	0.001	3.42	0.161	1	0.007	8.49	0.026	1	0.004	4.77	0.071
gs–7MAP	1	0.000	0.30	0.623	1	0.000	0.69	0.438	1	0.000	0.02	0.901
gs–8MAP	1	0.000	2.03	0.249	1	0.001	3.01	0.133	1	0.001	4.84	0.070
gs–9MAP	1	0.003	0.73	0.455	1	0.000	0.21	0.665	1	0.004	2.37	0.174
gs–12MAP	1	0.016	2.66	0.201	1	0.020	2.86	0.142	1	0.001	0.13	0.726
Tr–6MAP	1	0.767	52.0	0.005	1	1.078	9.60	0.021	1	0.021	0.19	0.678
Tr –7MAP	1	0.371	0.63	0.485	1	0.002	0.03	0.872	1	0.001	0.01	0.930
Tr –8MAP	1	1.528	1.91	0.261	1	0.587	3.11	0.128	1	0.892	4.72	0.072
Tr –9MAP	1	0.640	0.98	0.395	1	0.281	0.68	0.442	1	0.475	1.15	0.325
Tr–12MAP	1	2.080	1.17	0.357	1	1.605	1.24	0.308	1	0.019	0.01	0.906
WUE–6MAP	1	0.463	9.55	0.053	1	0.036	0.65	0.450	1	0.000	0.00	0.958
WUE–7MAP	1	0.910	0.48	0.538	1	0.081	0.23	0.645	1	0.485	1.40	0.281
WUE–8MAP	1	0.675	2.34	0.223	1	0.092	0.44	0.530	1	0.182	0.87	0.386
WUE–9MAP	1	1.995	9.68	0.052	1	0.513	2.41	0.171	1	0.867	4.08	0.089
WUE–12MAP	1	0.876	0.92	0.408	1	1.265	4.24	0.085	1	0.583	1.96	0.211
**Leaf**												
RS–6MAP	1	230.8	1.90	0.262	1	37.39	0.42	0.540	1	530.8	5.97	0.050
RS–7MAP	1	543.2	2.63	0.203	1	152.4	7.37	0.034	1	95.0	4.59	0.075
RS–8MAP	1	0.010	0.00	0.976	1	140.4	0.82	0.400	1	28.51	0.17	0.697
RS–9MAP	1	35.07	0.35	0.595	1	0.965	0.01	0.935	1	7.385	0.05	0.822
RS–12MAP	1	604.0	1.40	0.322	1	196.4	4.30	0.083	1	71.95	1.57	0.256
NRS –6MAP	1	1788	14.6	0.031	1	1169	15.7	0.007	1	76.91	76.91	1.030
NRS–7MAP	1	95.45	0.19	0.692	1	589.5	6.81	0.040	1	852.0	9.84	0.020
NRS–8MAP	1	807.5	0.82	0.431	1	119.3	1.26	0.305	1	3.376	0.04	0.856
NRS–9MAP	1	70.01	4.61	0.120	1	7.303	0.20	0.672	1	58.86	1.60	0.253
NRS–12MAP	1	83.44	0.11	0.760	1	130.1	0.79	0.407	1	713.6	4.35	0.082
TS–6MAP	1	734.1	1.94	0.258	1	788.4	6.46	0.044	1	203.7	1.67	0.243

Parameter	Water condition (W)				Cassava genotype (G)				W x G			
	df	MS	*F*–test	*P*–value	*df*	MS	*F*–test	*P*–value	*df*	MS	*F*–test	*P*–value
TS–7MAP	1	1094	10.5	0.047	1	142.3	1.30	0.298	1	377.8	3.44	0.113
TS–8MAP	1	813	0.69	0.468	1	518.5	15.13	0.008	1	12.23	0.36	0.572
TS–9MAP	1	204	3.86	0.144	1	2.916	0.01	0.916	1	24.67	0.10	0.761
TS–12MAP	1	238.4	5.06	0.109	1	646.3	4.14	0.088	1	332.4	2.13	0.194
Starch–6MAP	1	55.4	0.25	0.653	1	19.80	0.24	0.642	1	45.22	0.55	0.488
Starch–7MAP	1	37.3	1.08	0.375	1	1.816	0.04	0.850	1	77.74	1.66	0.245
Starch–8MAP	1	24.0	0.05	0.838	1	573.0	1.42	0.278	1	238.6	0.59	0.470
Starch–9MAP	1	115.6	0.27	0.641	1	5.784	0.04	0.850	1	249.4	1.67	0.244
Starch–12MAP	1	7.04	0.37	0.585	1	4.410	0.41	0.547	1	23.37	2.15	0.192
**Stem**												
RS–6MAP	1	3.036	0.03	0.865	1	13.15	0.18	0.682	1	85.97	1.20	0.314
RS–7MAP	1	21.55	2.85	0.189	1	3.600	0.85	0.391	1	8.895	2.11	0.196
RS–8MAP	1	14.23	7.16	0.075	1	5.844	0.58	0.476	1	3.506	0.35	0.577
RS–9MAP	1	8.13	2.37	0.221	1	17.11	3.82	0.098	1	2.665	0.59	0.469
RS–12MAP	1	539.7	16.6	0.026	1	0.095	0.00	0.970	1	8.19	0.13	0.727
NRS –6MAP	1	967.9	8.59	0.061	1	5.534	0.04	0.854	1	8.483	0.06	0.820
NRS–7MAP	1	0.135	0.01	0.920	1	23.54	0.29	0.608	1	14.00	0.17	0.691
NRS–8MAP	1	17.24	0.41	0.569	1	290.4	14.21	0.009	1	69.09	3.38	0.115
NRS–9MAP	1	788.3	38.4	0.008	1	19.16	2.38	0.174	1	137.6	17.08	0.006
NRS–12MAP	1	231.4	0.27	0.638	1	1501	4.55	0.076	1	690.7	2.09	0.198
TS –6MAP	1	1079	74.8	0.003	1	35.7	0.09	0.775	1	148.5	0.37	0.566
TS–7MAP	1	25.10	0.71	0.462	1	45.63	0.42	0.540	1	0.585	0.01	0.943
TS–8MAP	1	0.143	0.00	0.963	1	213.8	7.13	0.037	1	103.7	3.46	0.112
TS–9MAP	1	636.3	20.03	0.020	1	72.6	17.32	0.005	1	101.9	24.29	0.002
TS–12MAP	1	1477	2.47	0.214	1	1478	4.74	0.072	1	849.5	2.72	0.150
Starch–6MAP	1	4741	4.56	0.122	1	2218	7.33	0.035	1	1030	0.34	0.580
Starch –7MAP	1	1006	28.8	0.012	1	14253	9.06	0.023	1	1605	1.02	0.351
Starch –8MAP	1	77.31	0.05	0.832	1	174.9	0.04	0.839	1	2350	0.60	0.466

Parameter	Water condition (W)				Cassava genotype (G)				W x G			
	df	MS	*F*–test	*P*–value	*df*	MS	*F*–test	*P*–value	*df*	MS	*F*–test	*P*–value
Starch –9MAP	1	19187	8.04	0.065	1	5442.0	20.74	0.0039	1	1606.4	6.12	0.0482
Starch –12MAP	1	1233	0.09	0.788	1	2157.8	0.29	0.6085	1	3774.6	0.51	0.5018
**Tuber**												
RS–6MAP	1	36.000	0.18	0.697	1	52.562	35.13	0.0010	1	1.690	1.13	0.3288
RS–7MAP	1	1270.92	18.22	0.023	1	4.00	0.37	0.5667	1	18.92	1.74	0.2356
RS–8MAP	1	19.3600	1.48	0.310	1	6.0025	0.23	0.647	1	27.5625	1.06	0.3427
RS–9MAP	1	74.822	1.30	0.337	1	12.250	0.13	0.7325	1	214.622	2.25	0.1846
RS–12MAP	1	0.640	0.00	0.958	1	0.902	0.01	0.9108	1	249.640	3.78	0.0999
NRS –6MAP	1	147.016	46.81	0.006	1	553.426	8.65	0.0259	1	84.181	1.32	0.2950
NRS–7MAP	1	1283.43	3.63	0.152	1	128.26	0.84	0.3950	1	58.14	0.38	0.5601
NRS–8MAP	1	122.656	0.49	0.533	1	12.076	0.10	0.7680	1	138.651	1.09	0.3358
NRS–9MAP	1	31.641	0.66	0.476	1	109.726	7.08	0.0375	1	6.126	0.40	0.5527
NRS–12MAP	1	57.76	0.41	0.568	1	4489.00	15.46	0.0077	1	1256.70	4.33	0.0827
TS –6MAP	1	329.422	1.37	0.326	1	268.960	4.11	0.0891	1	111.302	1.70	0.2401
TS–7MAP	1	5101.53	29.52	0.012	1	178.89	1.06	0.3433	1	143.40	0.85	0.3926
TS–8MAP	1	234.856	0.66	0.476	1	35.106	0.15	0.7141	1	289.851	1.22	0.3119
TS–9MAP	1	9.456	0.07	0.814	1	49.351	0.40	0.5502	1	148.231	1.20	0.3149
TS–12MAP	1	71.40	0.27	0.636	1	4369.21	45.48	0.0005	1	388.09	4.04	0.0912
Starch–6MAP	1	56477.5	15.26	0.029	1	8621.1	1.17	0.3203	1	22801.0	3.10	0.1286
Starch–7MAP	1	5606.27	1.00	0.391	1	2306.40	0.77	0.4128	1	3.90	0.00	0.9723
Starch–8MAP	1	212613	1.85	0.267	1	17135	0.13	0.7302	1	43077	0.33	0.5874
Starch–9MAP	1	98831.6	5.00	0.111	1	11626.2	1.16	0.3222	1	55.9	0.01	0.9428
Starch–12MAP	1	5464.9	0.45	0.550	1	3195.1	1.09	0.3363	1	768.7	0.26	0.6266
MDA–6MAP	1	0.000	0.47	0.542	1	0.000	3.91	0.095	1	0.000	0.04	0.839
MDA–7MAP	1	0.007	282	0.001	1	0.000	9.21	0.022	1	0.002	2.76	0.147
MDA–8MAP	1	0.036	472	0.000	1	0.002	6.11	0.048	1	0.000	0.99	0.357
MDA–9MAP	1	0.000	0.84	0.426	1	0.003	11.3	0.015	1	0.000	0.14	0.721
H_2O_2–6MAP	1	0.001	0.08	0.797	1	0.539	48.2	0.000	1	0.013	1.21	0.314

Parameter	Water condition (W)				Cassava genotype (G)				W x G			
	df	MS	*F*–test	*P*–value	*df*	MS	*F*–test	*P*–value	*df*	MS	*F*–test	*P*–value
H_2O_2–7MAP	1	0.436	158	0.001	1	1.219	68.9	0.000	1	0.278	15.7	0.007
H_2O_2–8MAP	1	1.424	17.0	0.025	1	0.864	198	0.000	1	0.107	24.7	0.002
H_2O_2–9MAP	1	0.000	0.02	0.907	1	0.761	148	0.000	1	0.008	1.64	0.247
CAT–6MAP	1	239.5	12.3	0.038	1	5057	80.2	0.000	1	154.8	2.46	0.168
CAT–7MAP	1	2825	119	0.001	1	5828	538	0.000	1	1416	130.8	0.000
CAT–8MAP	1	3656	2412	0.000	1	8625	291	0.000	1	76.71	2.60	0.158
CAT–9MAP	1	221	4.40	0.126	1	8799	276	0.000	1	59.78	1.88	0.219
POX–6MAP	1	0.00	0.72	0.457	1	0.001	0.13	0.733	1	0.000	0.00	0.946
POX–7MAP	1	0.02	2.66	0.201	1	0.004	1.35	0.290	1	0.001	0.34	0.580
POX–8MAP	1	0.79	17.9	0.024	1	0.067	19.6	0.004	1	0.152	44.1	0.000
POX–9MAP	1	0.01	5.46	0.101	1	0.007	58.7	0.000	1	0.001	14.8	0.008
APX–6MAP	1	21.0	1.51	0.306	1	1300	108	0.000	1	1.670	0.14	0.721
APX–7MAP	1	1633	384	0.000	1	468.6	5.70	0.054	1	75.00	0.91	0.376
APX–8MAP	1	7023	176	0.001	1	334.1	7.43	0.034	1	50.85	1.13	0.328
APX–9MAP	1	2.41	0.02	0.902	1	722.1	119	0.000	1	5.918	0.98	0.360
Leaf DW–6MAP	1	14.06	0.00	0.952	1	9555.06	5.56	0.0564	1	2525.06	1.47	0.2709
Leaf DW–7MAP	1	66435.1	17.03	0.025	1	33580.6	32.07	0.0013	1	58443.1	55.82	0.0003
Leaf DW–8MAP	1	3220.6	1.58	0.297	1	14101.6	3.82	0.0983	1	280.6	0.08	0.7919
Leaf DW–9MAP	1	13514.1	10.27	0.049	1	15067.6	5.00	0.0668	1	5967.6	1.98	0.2091
Leaf DW–12MAP	1	23785.7	27.33	0.013	1	652.1	0.60	0.4729	1	375.0	0.35	0.5819
Petiole DW–6MAP	1	351.562	3.41	0.162	1	162.562	2.67	0.1534	1	52.562	0.86	0.3887
Petiole DW–7MAP	1	2756.25	7.70	0.069	1	2500.00	7.08	0.0375	1	2601.00	7.36	0.0350
Petiole DW–8MAP	1	20.25	0.41	0.567	1	4096.00	28.22	0.0018	1	1521.00	10.48	0.0178
Petiole DW–9MAP	1	1463.06	6.07	0.090	1	1463.06	3.83	0.0982	1	663.06	1.73	0.2359
Petiole DW–12MAP	1	1332.25	19.96	0.020	1	56.25	1.33	0.2927	1	4.00	0.09	0.7688
Stem DW–6MAP	1	10816	1.02	0.386	1	141752	4.48	0.0786	1	7310	0.23	0.6478
Stem DW–7MAP	1	738311	16.61	0.026	1	1312743	37.49	0.0009	1	697643	19.92	0.0043
Stem DW–8MAP	1	318660	2.76	0.195	1	1496952	16.43	0.0067	1	552	0.01	0.9405

Parameter	Water condition (W)				Cassava genotype (G)				W x G			
	df	MS	*F*–test	*P*–value	*df*	MS	*F*–test	*P*–value	*df*	MS	*F*–test	*P*–value
Stem DW–9MAP	1	36150	47.6	0.006	1	798789	6.58	0.0427	1	548711	4.52	0.0777
Stem DW–12MAP	1	11891	48.7	0.006	1	372710	3.91	0.0955	1	87912	0.92	0.3742
Root DW–6MAP	1	225.75	7.66	0.069	1	0.456	0.07	0.7972	1	79.656	12.62	0.0120
Root DW–7MAP	1	15.602	0.47	0.541	1	232.562	12.20	0.0129	1	19.803	1.04	0.3473
Root DW–8MAP	1	91.202	8.77	0.059	1	138.062	67.87	0.0002	1	81.902	40.26	0.0007
Root DW–9MAP	1	9.151	1.62	0.293	1	132.826	42.03	0.0006	1	0.106	0.03	0.8610
Root DW–12MAP	1	116.1	79.9	0.003	1	58.141	33.48	0.0012	1	34.516	19.88	0.0043
Tuber DW–6MAP	1	0.000	0.00	1.000	1	10201.0	0.43	0.5362	1	17030.3	0.72	0.4292
Tuber DW–7MAP	1	392189	9.75	0.052	1	730598	9.34	0.0223	1	775721	9.92	0.0198
Tuber DW–8MAP	1	6704216	3.05	0.179	1	1639040	1.77	0.2318	1	596370	0.64	0.4529
Tuber DW–9MAP	1	259845	2.57	0.207	1	855163	6.16	0.0477	1	630039	4.54	0.0771
Tuber DW–12MAP	1	8176740	55.2	0.005	1	1892	0.00	0.9505	1	263169	0.58	0.4739
Starch content–6MAP	1	27426.3	1.19	0.354	1	473.0	0.12	0.7402	1	684.9	0.17	0.6906
Starch content –7MAP	1	143829	7.70	0.069	1	95424	8.03	0.0298	1	165131	13.90	0.0098
Starch content –8MAP	1	300983	0.92	0.409	1	854259	1.62	0.250	1	812562	1.54	0.261
Starch content –9MAP	1	20626	0.14	0.734	1	298831	2.63	0.155	1	308682	2.72	0.1503
Starch content –12MAP	1	1291701	27.9	0.013	1	6551	0.11	0.747	1	34778	0.60	0.4670
Total DW–6MAP	1	29756	0.15	0.728	1	448900	4.61	0.075	1	23409	0.24	0.6412
Total DW–7MAP	1	2843439	9.91	0.051	1	457991	2.00	0.207	1	3441953	15.00	0.0082
Total DW–8MAP	1	3870073	1.31	0.335	1	9851	0.01	0.934	1	765188	0.57	0.4803
Total DW–9MAP	1	1485352	12.0	0.040	1	11289	0.03	0.877	1	2806463	6.48	0.0437
Total DW–12MAP	1	0.0000	58.8	0.004	1	500556	0.49	0.508	1	685584	0.68	0.4422
